# Landomycins as glutathione-depleting agents and natural fluorescent probes for cellular Michael adduct-dependent quinone metabolism

**DOI:** 10.1038/s42004-021-00600-4

**Published:** 2021-11-25

**Authors:** Alessio Terenzi, Mery La Franca, Sushilla van Schoonhoven, Rostyslav Panchuk, Álvaro Martínez, Petra Heffeter, Redding Gober, Christine Pirker, Petra Vician, Christian R. Kowol, Rostyslav Stoika, Luca Salassa, Jürgen Rohr, Walter Berger

**Affiliations:** 1 Department of Biological, Chemical and Pharmaceutical Sciences and Technologies, University of Palermo, Viale delle Scienze, Ed. 17, 90128 Palermo, Italy; 2 Institute of Cancer Research and Comprehensive Cancer Center, Medical University Vienna, Spitalgasse 23, 1090 Vienna, Austria; 3 Department of Regulation of Cell Proliferation and Apoptosis, Institute of Cell Biology, Drahomanov St., 14/16, Lviv, 79005 Ukraine; 4 Donostia International Physics Center and Polimero eta Material Aurreratuak: Fisika, Kimika eta Teknologia, Kimika Fakultatea, Euskal Herriko Unibertsitatea UPV/EHU, Paseo Manuel de Lardizabal 4, Donostia, 20018 Spain; 5 Research Cluster “Translational Cancer Therapy Research”, University of Vienna and Medical University of Vienna, Vienna, Austria; 6 College of Pharmacy, University of Kentucky, South Limestone Str. 789, Lexington, 40536-0596 USA; 7 Institute of Inorganic Chemistry, Faculty of Chemistry, University of Vienna, Waehringer Straße 42, 1090 Vienna, Austria; 8 Ikerbasque, Basque Foundation for Science, Bilbao, 48011 Spain

**Keywords:** Mechanism of action, Small molecules

## Abstract

Landomycins are angucyclines with promising antineoplastic activity produced by *Streptomyces* bacteria. The aglycone landomycinone is the distinctive core, while the oligosaccharide chain differs within derivatives. Herein, we report that landomycins spontaneously form Michael adducts with biothiols, including reduced cysteine and glutathione, both cell-free or intracellularly involving the benz[a]anthraquinone moiety of landomycinone. While landomycins generally do not display emissive properties, the respective Michael adducts exerted intense blue fluorescence in a glycosidic chain-dependent manner. This allowed label-free tracking of the short-lived nature of the mono-SH-adduct followed by oxygen-dependent evolution with addition of another SH-group. Accordingly, hypoxia distinctly stabilized the fluorescent mono-adduct. While extracellular adduct formation completely blocked the cytotoxic activity of landomycins, intracellularly it led to massively decreased reduced glutathione levels. Accordingly, landomycin E strongly synergized with glutathione-depleting agents like menadione but exerted reduced activity under hypoxia. Summarizing, landomycins represent natural glutathione-depleting agents and fluorescence probes for intracellular anthraquinone-based angucycline metabolism.

## Abstract (web summary)

Landomycins are promising anti-cancer agents with the potential to bypass common resistance pathways, but their mechanism of action remains incompletely understood. Here a mechanism is proposed involving intracellular sequential thiol-Michael and oxidation reactions, which depletes endogenous thiols and induces reactive oxygen species formation.

## Introduction

**Fig. 1:**
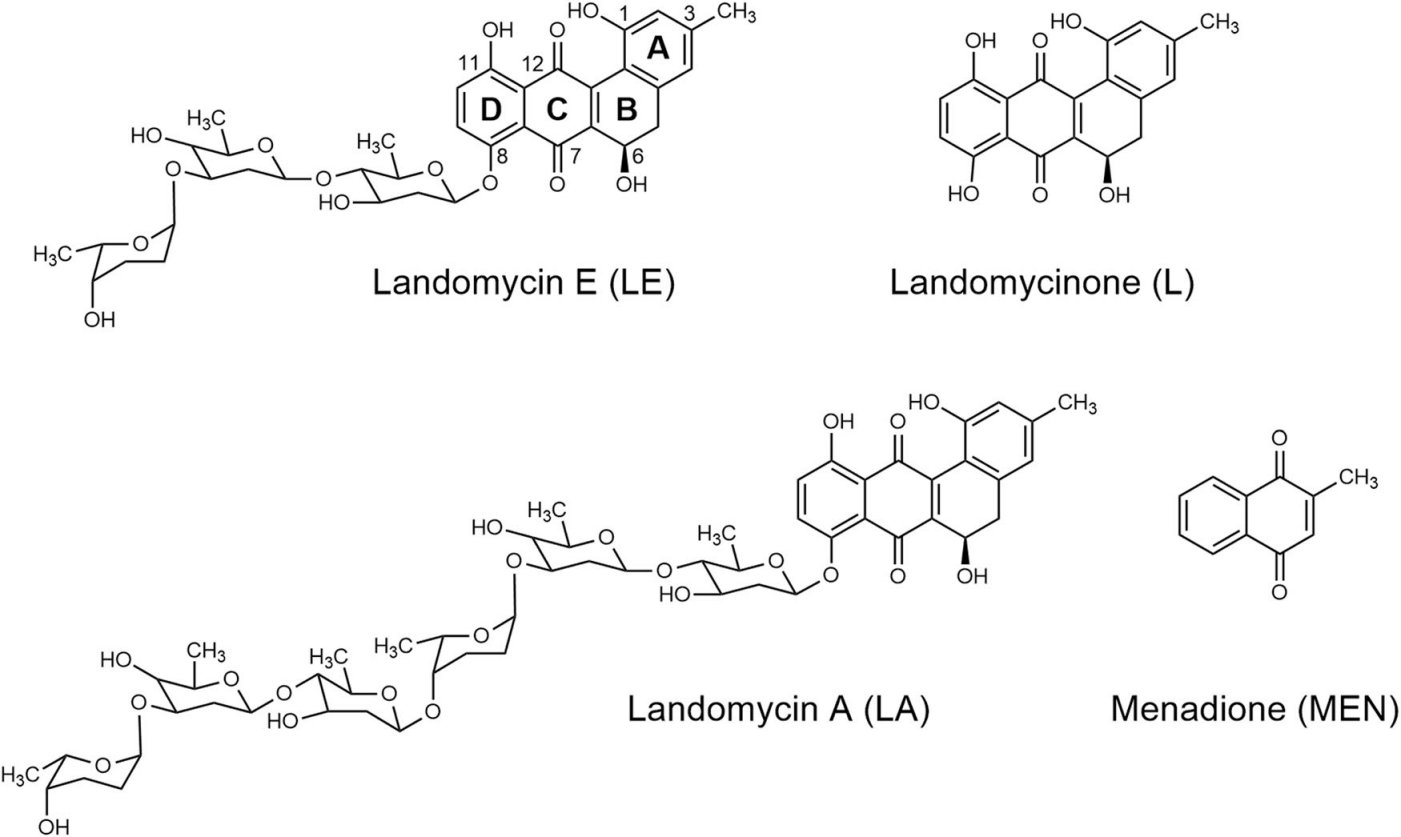
Chemical structure of the compounds used. Chemical structure of landomycin E (LE), landomycin A (LA), landomycinone (L) and menadione (MEN).

Angucyclines are a family of natural antibiotics produced by *Streptomyces* bacteria and were discovered following the success of tetracyclines and anthracyclines (e.g., daunorubicin and doxorubicin) both in broad medical use as antibiotics and anticancer compounds^1–3^. Similar to these well-known substance classes, angucyclines are characterized by a tetracyclic ring skeleton but assembled in a benz[a]anthracene system owning a distinctive angular structure, reflected in the name angu-cyclines^4,5^. This family of natural compounds represents one of the most bioactive polycyclic aromatic polyketides to date, characterized by promising antiviral, antibacterial and antitumor properties^5^. Among the different chemical scaffolds of angucyclines, landomycins are the most auspicious antineoplastic agents so far^5,6^. They are characterized by a benz[a]anthraquinone core bearing a nonaromatic B ring and a linear glycosidic chain of various length attached to the hydroxyl at position C8 (Fig. 1)^5–7^. Besides extraction and purification from bacterial cultures, strategies for total chemical synthesis of several landomycins have been developed^8–10^. Landomycin A (LA) and landomycin E (LE), containing six and three saccharide residues, respectively, are the most studied compounds of this family^3,7,11–13^. In particular, LE showed potent activity against numerous cancer cell models in vitro and demonstrated to be unaffected by resistance to structurally related anthracyclines (e.g., doxorubicin), used in clinics for the treatment of several malignancies^3,5,11^.

Despite these efforts, the mechanisms-of-action underlying the antineoplastic activity of angucyclines are not fully understood yet. Considering their structural similarities with doxorubicin, some anthraquinone-based angucyclines have been proposed to act in a similar way, including a direct DNA targeting (examples are jadomycin B and hedamycin)^14,15^. However, we have demonstrated that LE shows a different mechanism of action when compared with doxorubicin: it does not intercalate into DNA and induces massive mitochondrial dysfunction and membrane depolarization followed by apoptosis induction^11^. Rodriguez et al. reported on another anticancer angucycline, marmycin A, which acts differently from doxorubicin and accumulates in lysosomes^16^, further demonstrating the potential of this class of natural products in fighting cancers refractory to doxorubicin-based treatments.

In previous investigation, we have shown that landomycins exert potent anticancer activity by inducing apoptotic cell death as a consequence of mitochondrial damage^3,11^. As part of our continuing efforts toward the dissection of landomycins’ mechanism of action, we have recently elucidated that the intense activity of LE against leukemia cells (e.g., acute T-cell leukemia Jurkat cells) involved the generation of reactive oxygen species (ROS) and hydrogen peroxide. However, surprisingly, exposure of Jurkat cells to complex mixtures of diverse ROS scavengers only partly blocked the cytotoxic LE effects, while co-incubation with the reduced glutathione (GSH) precursor N-acetylcysteine (NAC) almost completely abrogated its activity^3^. Consequently, we hypothesized that NAC might have formed an adduct with LE extracellularly, probably leading to a reduced uptake of the drug^3^.

At closer inspection, landomycins indeed feature an anthraquinone moiety in their core structure (Fig. 1), which might be hypothesized to be a key player in landomycins’—and perhaps other angucyclines’—anticancer properties^17–19^. Quinones are known to exert their activity through ROS generation via redox cycling^20–22^. Some of these quinones are additionally Michael acceptors. Therefore, they can provoke cellular damage through covalent bonds with nucleophilic cysteinyl thiols^20,21,23^. Interesting examples of molecules with enhanced activity due to Michael adduct formation are epigallocatechin-3-gallate (EGCG, a major component of green tea) and 3,4-(±)-methylenedioxymethamphetamine (MDMA, the so-called recreation drug ecstasy), whose metabolites are quinones that are able to interact with thiols^20,21,24^. It was also shown that the SCH_3_ group of the angucycline antibiotic urdamycin E derives from methanthiol Michael addition to urdamycin A, a compound similar to the landomycins^25^.

Herein, we present a detailed study on the relationship between the anticancer activity of landomycins with a focus on LE and its capability to form Michael adducts with biothiols. Conjugation with cysteine (Cys) or reduced GSH induces a glycosidic chain-dependent fluorescence pattern on the nonemissive LE molecule allowing to follow the fate of landomycin both cell-free and in cells by fluorescence-based techniques. Moreover, this is the first report so far directly connecting the activity of landomycin antibiotics to quinone-conjugation processing, possibly casting light on the mode of action of all benz[a]anthraquinone-based angucyclines. Ultimately, we suggest novel therapeutic combinations of landomycins with other GSH-depleting and ROS-producing agents.

## Results and discussion

### Extracellular cysteinyl thiols protect cells from LE more efficiently than ROS scavenging

**Fig. 2:**
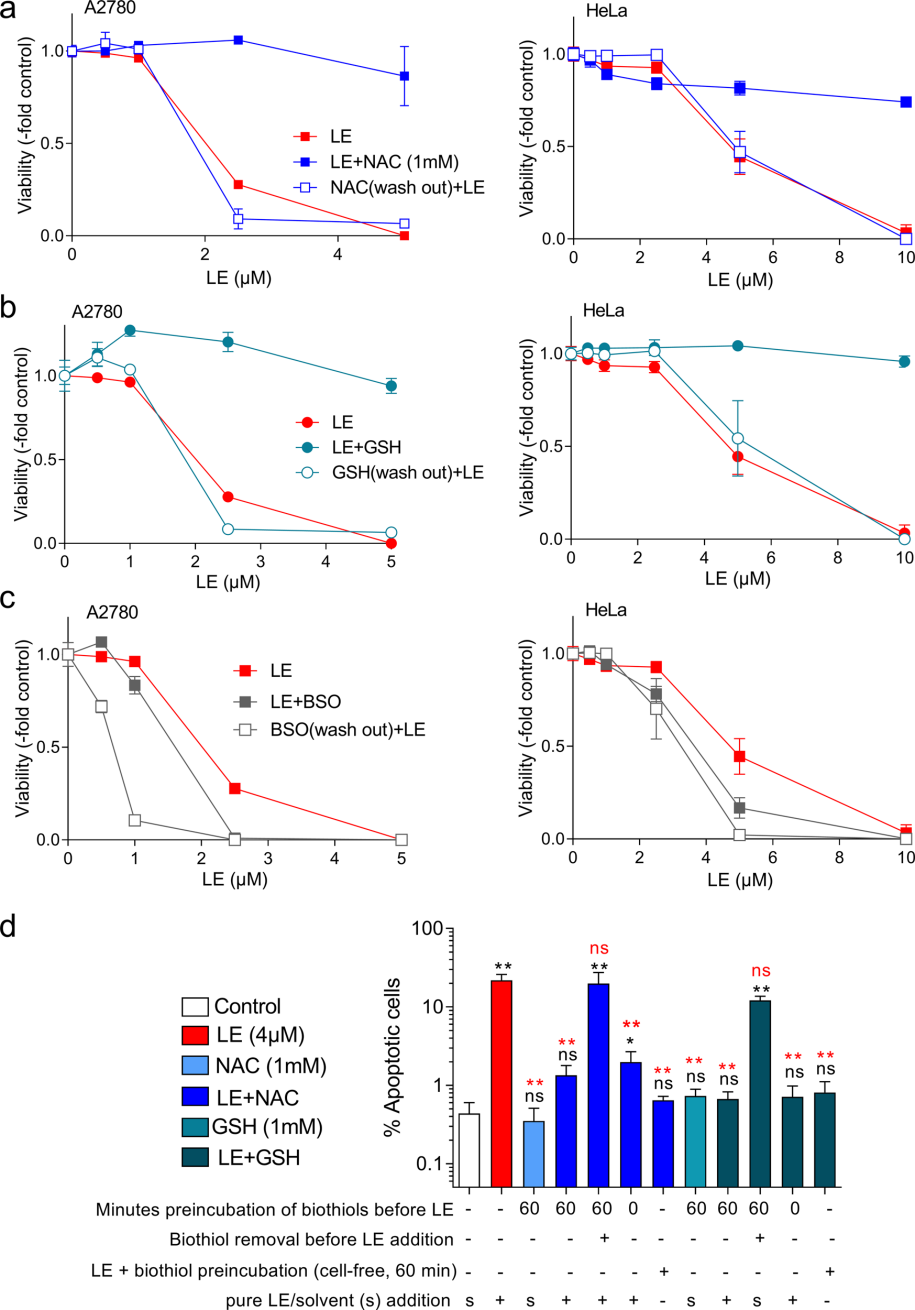
Impact of small biothiols and BSO on LE cytotoxicity. **a**, **b**, **c** A2780 (left panels) or HeLa (right panels) cells were exposed for 72 h to increasing concentrations of LE only or preincubated for 60 min with NAC (**a**), GSH (**b**), or BSO (**c**) before addition of LE. Viability was determined using MTT assay. Each data point represents the mean ± standard deviation (SD) of triplicate values from one representative experiment out of three delivering comparable results. **d** Jurkat leukemic cells were treated with LE and small biothiols as indicated and apoptotic cells were quantified by Annexin V–APC/PI flow cytometry. Mean ± SD from one experiment performed in triplicate is shown. Statistical differences between solvent control (s, white column) and treated samples were calculated with student’s *t*-test (*p < 0.05, **p < 0.01) and indicated by black asterisks above the respective columns. Statistical differences between LE single treatment (red column) and combined settings with NAC or GSH were calculated using two-way ANOVA (**p < 0.01) and are indicated by red asterisks. ns nonsignificant.

In order to better understand the mechanisms underlying the profound protective effect of NAC (or GSH) against LE cytotoxicity, we performed a series of intra- and extra-cellular drug exposure experiments by an MTT-based cell-viability assay^26–28^. As already reported for Jurkat cells before^3^, also exposure of A2780 cells to LE led to a complete loss of cell viability already at 5 µM, while co-incubation with 1 mM NAC or GSH (added to cells 1 h before LE) completely inhibited this cytotoxic effect. HeLa cells were more resistant against LE, but again, full protection from the massive cytotoxicity of 10 µM LE was observed by NAC/GSH coexposure (Fig. 2a, b). Accordingly, preincubation (24 h) with the glutathione synthesis inhibitor L-buthionine-(S,R)-sulfoximine (BSO) moderately but significantly sensitized against LE. This effect was, however, by far less potent as compared with the NAC-mediated protection against LE (Fig. 2c). Additionally, removal of NAC or GSH after 1 h preexposure, before addition of LE, abolished the protective effects observed when these cysteinyl thiols were kept in the cell culture medium during LE exposure (compare Fig. 2a, b). To confirm this interaction in another cell and assay background, we used leukemic Jurkat cells and detected LE-mediated apoptosis via flow cytometry by annexin-V staining. Indeed, preincubation (60 min) with both NAC and GSH significantly blocked cell death induction upon LE treatment (Fig. 2d, *p* < 0.01). Additionally, removal of both NAC and GSH prior to LE exposure led to cytotoxic effects similar to LE single treatment. Interestingly, cell-free preincubation of LE with biothiols (NAC or GSH for 60 min) before addition to cell culture, as well as concurrent addition of the thiols with LE directly onto the cells, both completely abrogated LE-mediated apoptosis induction. This indicates that upregulation of intracellular GSH concentrations by the short NAC preincubation is not sufficient to efficiently protect cells from LE-mediated cytotoxicity and that obviously a direct interaction between NAC and LE, most probably extracellularly, needs to be considered.

### LE spontaneously forms an adduct with biothiols

**Fig. 3:**
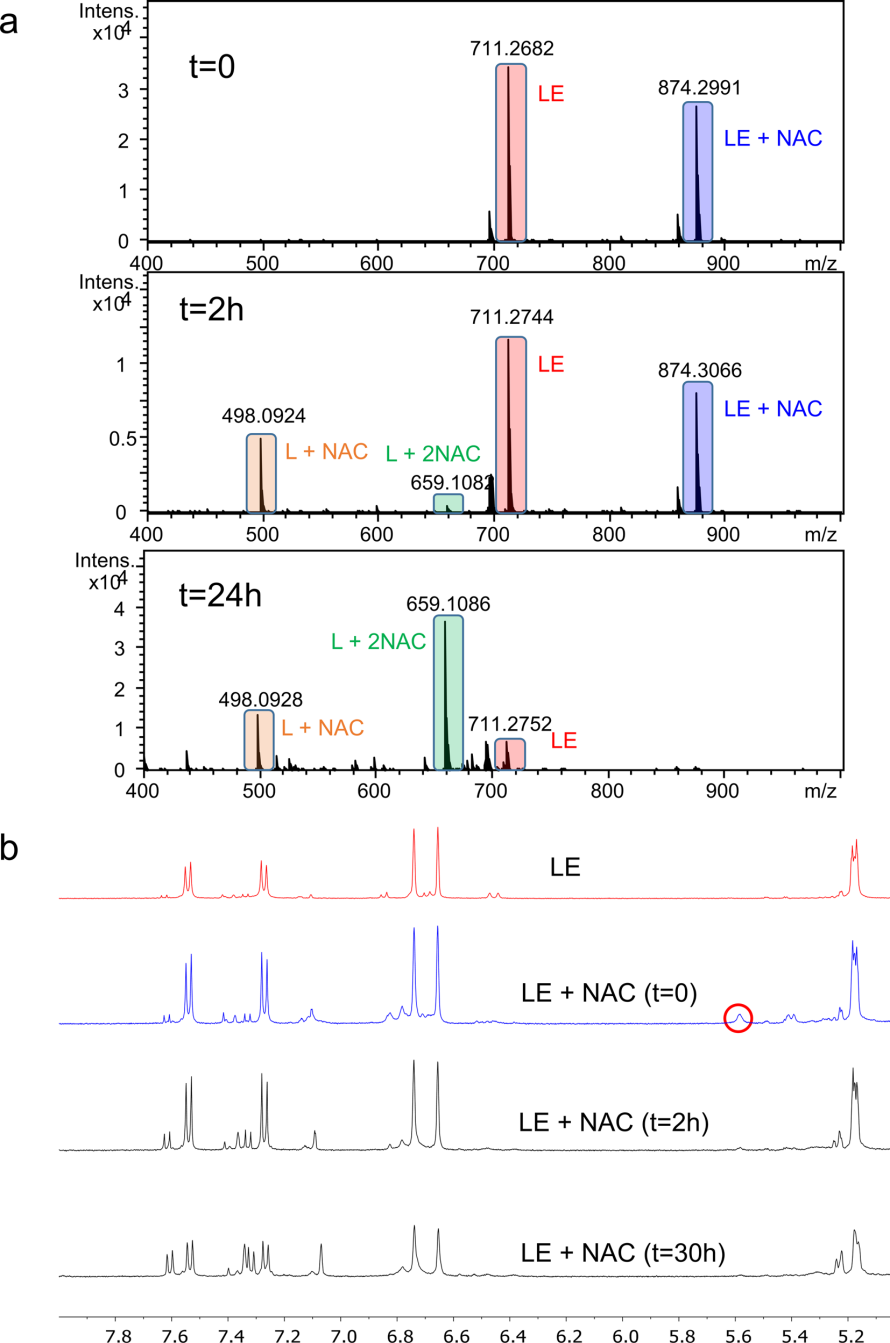
Mass spectrometry and NMR studies of LE and NAC interaction. **a** Time-dependent ESI–TOF mass spectra of LE (0.2 mM) mixed with NAC (0.4 mM) in H_2_O with 20% MeOH. The peaks corresponding to the different species are highlighted with boxes of different colors: LE (red box), LE + NAC (blue box), L + NAC (orange box), and L + 2NAC (green box). **b** ^1^H-NMR in CD_3_OD of LE (7 mM, in red) and of LE (7 mM) mixed with NAC (10 mM) at different time points as indicated in the legend.

As mentioned above, the chemical structure of landomycins features a benz[*a*]anthraquinone moiety (Fig. 1). In selected cases, quinones are known to undergo spontaneous conjugation with biothiols. Consequently, we performed high resolution mass spectrometry (ESI-TOF) experiments to elucidate whether Michael adduct formation takes place between LE and cysteinyl thiols under cell-free conditions. Immediately after mixing the quinone LE and the nucleophilic thiol NAC, the adduct LE + NAC was clearly detectable (peak at m/z = 874.3, Fig. 3a and Supplementary Fig. 1), demonstrating a strong and fast interaction between the two chemical entities. Interestingly, the peak at 711.3 corresponding to the m/z value of LE is followed by another one at 713.3 with the isotope-distribution pattern of LE in its reduced form (i.e., hydroquinone, two additional protons in mass spectrum), reasonably suggesting that the thiol, besides forming the adduct, also exerts a reductive action (Supplementary Fig. 1). We decided to follow the kinetics of the adduct formation via mass spectrometry at different time points (compare Fig. 3a, Supplementary Figs. 2 and 3). Interestingly, after two hours, in addition to the LE + NAC adduct, two new species were detectable: one in which the adduct loses the glycosidic chain, leading to the aglycone L in combination with NAC (L + NAC) and another one, less abundant, in which L is combined with two NAC molecules (L + 2NAC). Indeed, once the glycosidic chain is lost, the aglycone is ready to accept a second molecule of NAC (e.g., in position 9). The formation of bis- or even multiple-thiol Michael adducts has been reported for a number of quinones^29–31^. The main adduct LE + NAC is consumed overtime (4, 8, and 24 h), and after 24 h, is no longer detectable in favor of the L + NAC and L + 2NAC species, the latter becoming the most abundant.

**Fig. 4:**
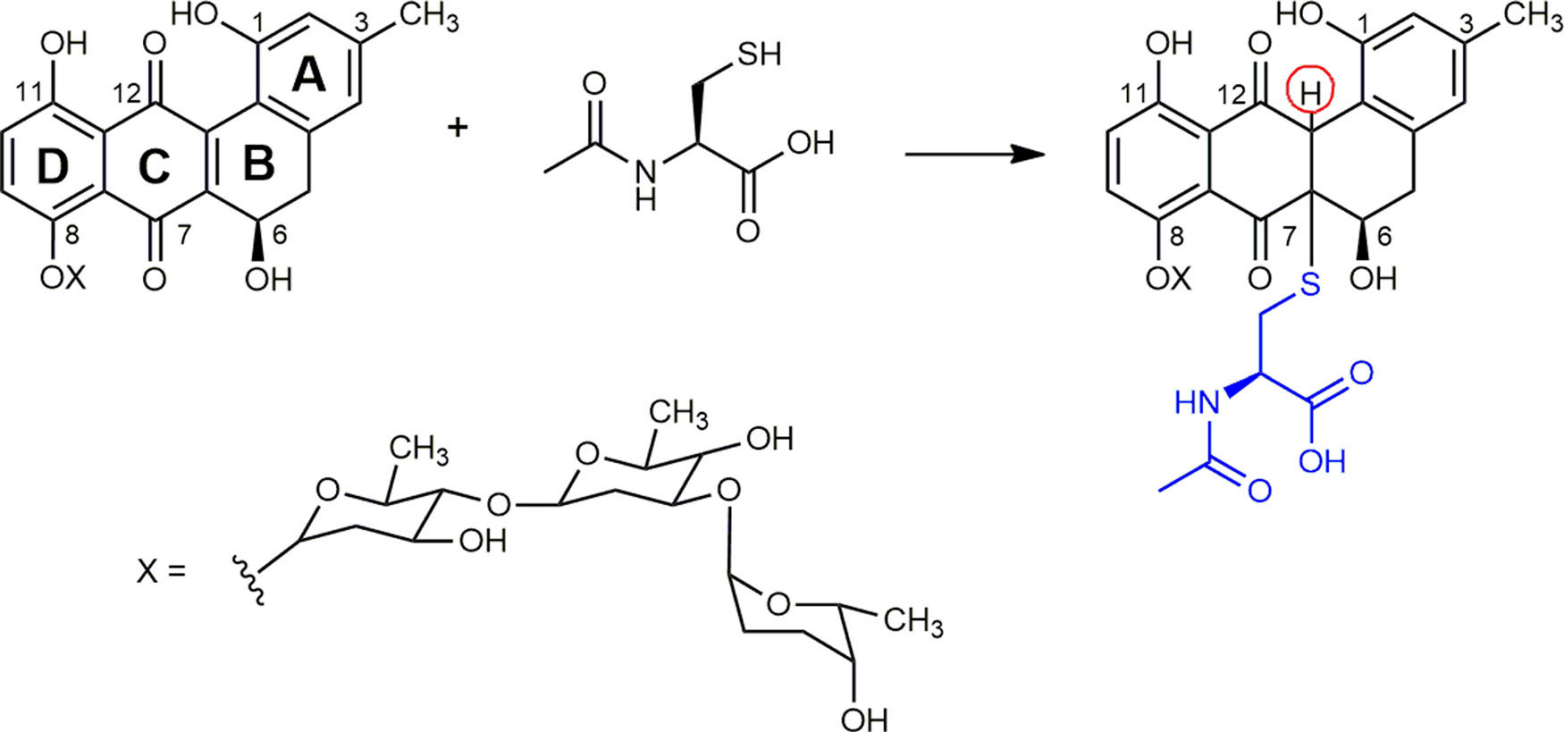
LE–NAC adduct formation. Reaction scheme of LE and NAC and proposed structure of the formed adduct.

The adduct formation, together with the following chemical evolution, could be confirmed by NMR spectroscopy (Fig. 3b and Supplementary Fig. 4). NMR of LE alone (red line, Fig. 3b; for full ^1^H and ^13^C NMR spectra see Supplementary Fig. 5a) showed a multiplet at 5.17 corresponding to proton at position 6, two singlets at 6.66 and 6.74 ppm, corresponding to the aromatic protons at positions 2 and 4 of ring A, and two doublets at 7.27 and 7.54, corresponding to the aromatic protons at positions 9 and 10 of ring D (see Fig. 4). In addition, a set of signals corresponding to approximately 5% of the aglycone L was observed^8^.

After the addition of NAC to a LE solution (*t* = 0, blue line, Fig. 3b), a new set of peaks emerged. The signal at 5.58 ppm (red circle, Fig. 3b) can be assigned to the new proton between rings B and C of the proposed adduct structure (red filled circle, Fig. 4). Upon formation of the adduct, the signal corresponding to proton 6, originally at 5.17 ppm, was downshifted up to 5.40 ppm while maintaining its multiplicity. Among all the possible regioisomers of the adduct, this suggests that the thiol reacts with the activated double bond between rings B and C rather than with ring D. Additionally, if the position of the thiolate and the new proton would be the opposite as those depicted in Fig. 4, we would expect higher complexity in both the above-mentioned signals due to the additional coupling between the new proton and the one in position 6 with a smaller shift of the latter.

Over time (*t* = 2 and 30 h, black lines, Fig. 3b), the peaks at 5.40 and 5.58 ppm completely disappeared, while there was a gradual decrease of the two singlets at 6.66 and 6.74 ppm and of the two doublets at 7.27 and 7.54 ppm. At the same time, new singlets at 7.07 and 7.35 ppm and doublets at 7.32 and 7.70 ppm appeared. Overall, these results point toward the existence of a relatively short-lived monoadduct species and an underlying process most likely leading to the L + 2NAC species observed in the mass experiment.

Control NMR experiments confirmed that LE without addition of cysteinyl thiols is stable in solution (Supplementary Fig. 5b).

High-resolution mass spectrometry experiments clearly indicated that GSH and Cys produced similar Michael adduct species directly after mixing (Supplementary Figs. 6 and 7), although the adduct LE + NAC under the same conditions was relatively more abundant. The interaction of LE and GSH over time (Supplementary Figs. 8 and 9) followed the same path as the one of LE and NAC, including the loss of the glycosidic chain and the reaction with a second GSH molecule.

### The LE Michael adduct exhibits specific fluorescence properties

**Fig. 5:**
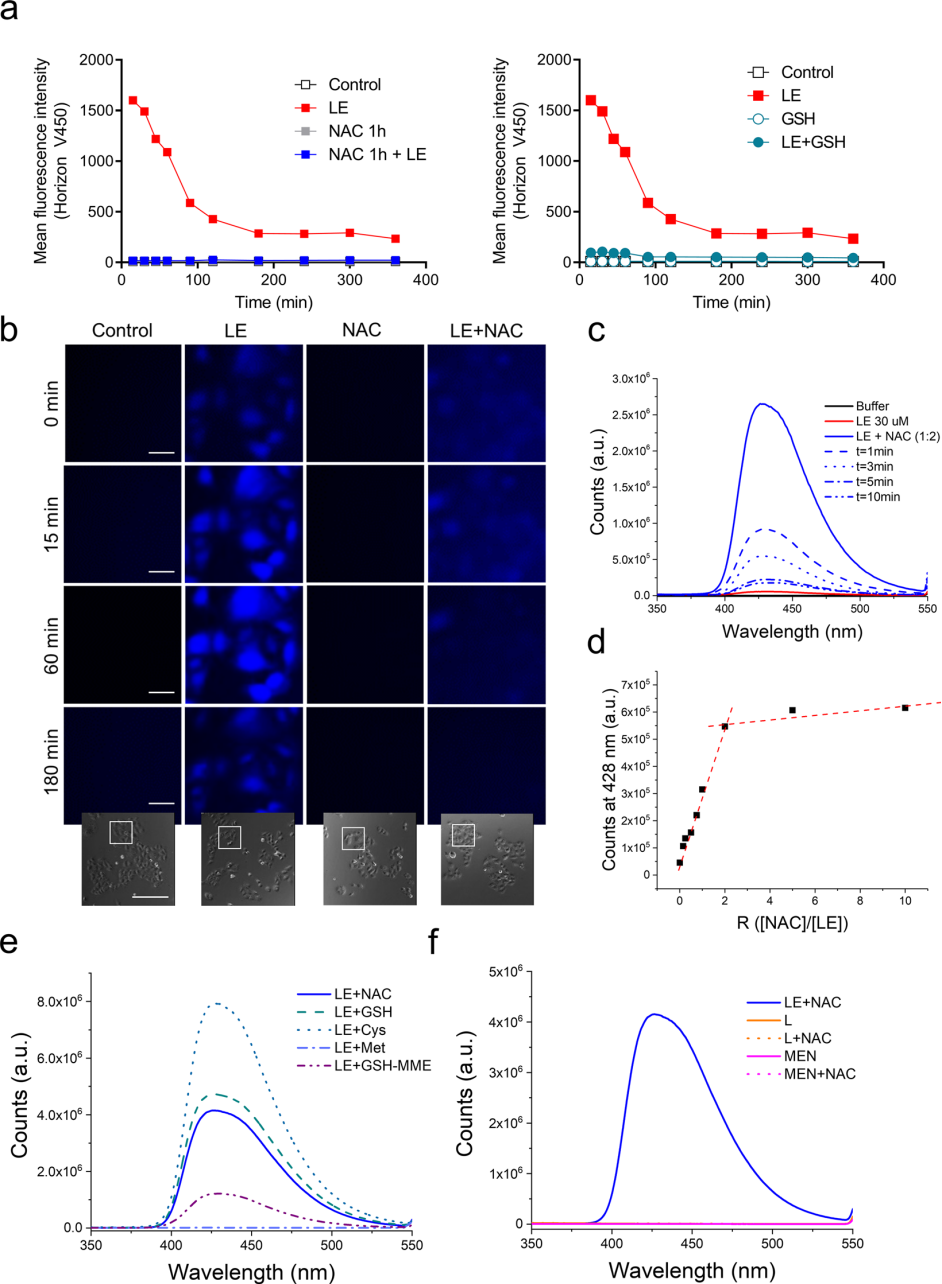
Landomycin E fluorescent properties upon Michael adduct formation. **a** Intracellular fluorescence of LE exposed Jurkat cells was detected by flow cytometry. Cells were treated with 4 µM LE-alone or after preincubation with the cysteinyl thiols NAC (1 mM, left panel) or GSH (1 mM, right panel). Intracellular fluorescence was quantified at indicated time points by flow cytometry (Horizon V450 (emission 448 nm)). **b** LE-induced intracellular fluorescence of MG63 osteosarcoma cells was followed over time by live-cell imaging (DAPI channel) after treatment with 4 µM LE with or without 1 mM NAC pre/coincubation (1 h). Scale bar indicates 10 µm (fluorescence images) and 100 µm (phase-contrast insets). The small phase-contrast images at the bottom, taken at the start of the experiment (time point 0), indicate the cell groups shown enlarged in the respective upper fluorescence panels. **c** Fluorescence profile of LE alone (red solid line) and in combination with NAC (*λ*_exc_ = 280 nm). Spectra of the same solution have been recorded immediately after mixing LE with NAC (blue solid line) and after the time points indicated in the legend (blue dashed and dotted lines). Slits width: 5 nm/5 nm. **d** Plot of LE emission in combination with NAC (*λ*_exc_ = 280 nm, *λ*_em_ = 428 nm) at different concentrations. **e** Fluorescence profile of 40 μM LE in combination with 200 μM NAC (blue solid line), GSH (green dashed line), Cys (sea green dotted line), GSH–MME (violet dash–dot–dot line), and Met (light blue dash–dot line). Slit width: 2 nm/5 nm. **f** Fluorescence profile of 15 μM L (orange solid line) and 30 μM menadione (MEN, fuchsia solid line) in combination with 60 μM NAC (corresponding dotted lines). For comparison, the fluorescence profile of 40 μM LE in combination with 200 μM NAC is depicted as blue solid line. *λ*_exc_ = 280 nm; slit width: 5 nm/5 nm; Buffer: Tris-HCl 50 mM, pH = 7.4 for **b**, **c**, **d**, and **e**.

Next, we aimed to address the question whether LE adduct formation might also take place in living cells. Cell-free LE in solution is basically nonfluorescent as proven by its full fluorescence excitation–emission 3D landscape in Tris-HCl buffer (Supplementary Fig. 10a). Unexpectedly, however, LE-exposed human-immortalized T-lymphocyte Jurkat cells were characterized by a surprisingly strong but relatively short-lived fluorescent signal in the blue Horizon V450 channel (*λ*_exc_ = 404 nm, *λ*_em_ = 448 nm) of the flow cytometer (Fig. 5a). LE-associated fluorescence was confirmed by live-cell fluorescence microscopy, performed as published^32^, and localized to the cytoplasm with enhanced accumulation in the cell nucleus of human LN229 glioblastoma and MG63 osteosarcoma cells (Supplementary Fig. 10b and Fig. 5b, respectively). Interestingly, co-incubation with either NAC or GSH strongly reduced intracellular signals but instead resulted in a diffuse blue fluorescence in the extracellular space (Fig. 5b). These findings indicated that the LE–thiol adduct is fluorescent and poorly internalized by cells. In contrast, cell-free preincubation of LE with the cysteinyl thiols for 1 h completely abrogated the cell-associated fluorescent signal (Fig. 5a). In addition, the blue fluorescence signal detected by flow cytometry or under the microscope was transient and progressively decreased, especially during the first two hours of incubation (Fig. 5a, b). Together, these data strongly suggest that the observed Michael adduct of LE and its chemical evolution might also take place in the living cells and underlie the strong cellular fluorescence signals.

Accordingly, we decided to study the fluorescent properties of LE without and with cysteinyl thiols in solution. When excited at 280 nm, LE alone was again essentially non-emissive (Fig. 5c, solid red line). However, immediately after mixing LE with NAC, a dramatic emission enhancement at 428 nm was observed (Fig. 5c, solid blue line and Supplementary Fig. 11 for the excitation/emission profiles). The species responsible for this emission band was short lived, since the fluorescence signal decreased within 10 min (Fig. 5c, dashed and dotted blue lines).

To investigate photobleaching of the LE adduct as possible explanation for the short-lived nature of the fluorescence signal, we prepared different solutions of LE mixed with NAC and measured their emission after diverse incubation times (with the solutions carefully protected from external light sources) (Supplementary Fig. 12). The strong fluorescence signal at 428 nm was only produced by the freshly mixed solution at time point zero, while this signal was strongly quenched after an incubation time of only 10 min, demonstrating that the emission decrease was solely due to the chemical evolution of the LE-NAC adduct and not because of the irradiation of the sample (by the instrument or by external light). We then evaluated the effect of the NAC concentration on the LE fluorescence intensity. As shown in Fig. 5d and Supplementary Fig. 13, the emission band at 428 nm increased after addition of several NAC aliquots, until a plateau appeared when the ratio NAC/LE was equal to 2 (stoichiometry is indicated by the two crossing red lines in the graph, Fig. 5d). Nevertheless, considering the transient nature of the emission and the short life span of the LE–NAC adduct, we believe that LE–NAC is reasonably the emissive species, rather than its analog with two NAC moieties. Under the experimental condition used, the excess of NAC obviously served to produce the reduced form of LE, as indicated by mass experiments.

Biothiols GSH, Cys, and GSH derivatives like glutathione monoethyl ester (GSH-MME), interacting with LE in the same way as NAC, were also inducing a fluorescence burst as shown in Fig. 5e. Also, the LE–GSH and LE–Cys-associated fluorescence decreased over time, however, LE–Cys emission appeared to be more stable (Supplementary Fig. 14). The essential amino acid methionine (Met), lacking the –SH group, did not produce any fluorescence in combination with LE (Fig. 5e), indicating that the Michael adducts are, indeed, the fluorescent species.

Strikingly, the fluorescence generated by Michael addition resulted to be a distinctive property of LE. The similar arylating 1,4-naphthoquinone menadione (MEN), which in accordance with the literature^33^ and our mass spectrometry experiments (Supplementary Fig. 15) also forms an adduct with NAC, does not show any fluorescence emission when excited at different wavelengths, neither alone nor in combination with NAC (Fig. 5f).

LE and MEN, while comparable in their core quinone structure, differ in the presence of the glycosidic chain in the landomycin molecule (see Fig. 1). Consequently, the question arose whether the sugar could play an important role in the fluorescence burst. Accordingly, we measured the fluorescence profile of the landomycinone (L), which corresponds to the aglycone of the LE deprived of the glycosidic chain (see Fig. 1). Figure 5f shows that L, even when it forms an adduct with NAC as demonstrated by mass spectrometry (Supplementary Fig. 16), does not induce any fluorescence burst, indicating an important role of the sugars in the general fluorescence properties. This finding is corroborated by the blue emission produced, when NAC was added to LA (Supplementary Fig. 17a, a landomycin derivative containing six sugar units (see Fig. 1)). We wondered if sugars might play a role as simple electron donors, regardless of the fact that they are part of the LE structure. However, L did not show any fluorescence in the presence of NAC and three equivalents of glucose (Supplementary Fig. 17b), demonstrating that the mechanism behind the emission development is not trivial.

### Fluorescence and adduct formation in biological samples

In the cellular microenvironment and inside cells, there are, besides small molecules like Cys and GSH, multiple other proteins containing free -SH groups potentially able to attack electrophilic moieties through Michael addition. We analyzed LE fluorescence changes in combination with human serum albumin, which contains a free -SH group, or with cell culture medium containing or lacking fetal calf serum (FCS). In addition, interaction of LE with the complex biological matrix of a living cell was measured by spiking the compound into crude protein extracts from human cancer cells. The mixture of LE with pure human serum albumin in buffered solution did not induce the typical adduct-dependent fluorescence enhancement at 428 nm (Supplementary Fig. 18), but a quenching of the intrinsic albumin fluorescence at 300–350 nm was observed, indicating a possible interaction between the two molecules. Additionally, once NAC was added to the albumin–LE mixture, the LE–NAC fluorescence at 428 nm was present but distinctly reduced as compared with albumin-free conditions, strongly suggesting that LE is still but less accessible for adduct formation with cysteinyl thiols.

**Fig. 6:**
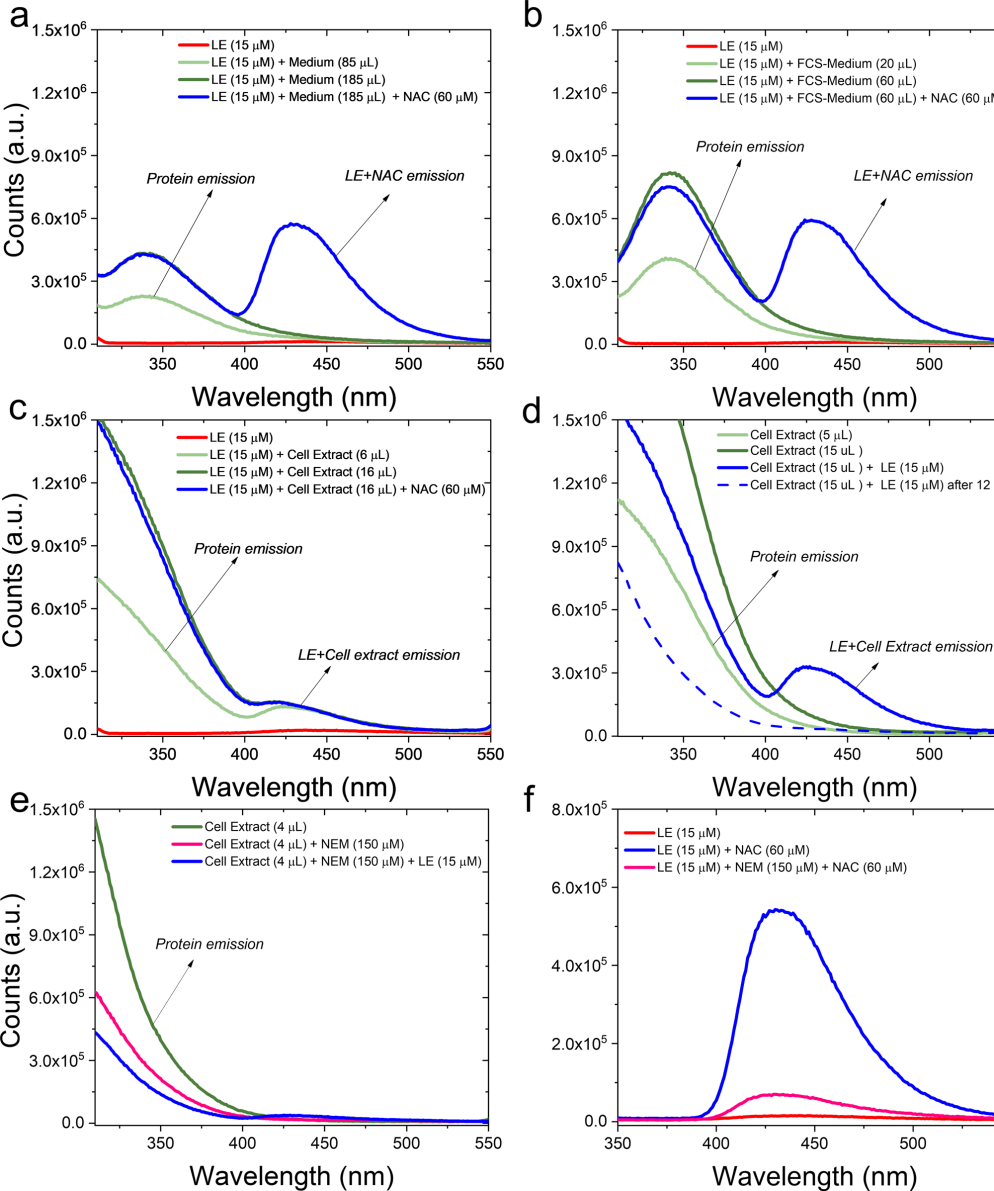
LE-related fluorescence in cell culture medium and cell cytosolic extracts. Fluorescence profile of (**a**) LE (red line) in combination with cell culture medium without (green and light-green lines) and with NAC (blue line); **b** LE (red line) in combination with cell culture medium supplemented with FCS without (green and light-green lines) and with NAC (blue line); **c** LE (red line) in combination with Hep3B cell extract (protein content: 3.7 μg/μl, diluted in 2 ml) without (green and light-green lines) and with NAC (blue line); **d** Hep3B cell extract (green and light green lines) in combination with LE (blue solid line), also after 12 h (blue dashed line); **e** Hep3B cell extract alone (green line), preincubated with NEM (fuchsia line) and after mixing the latter with LE (blue line); **f** LE (red line) in combination with NAC (blue line) and with NAC previously preincubated with NEM (fuchsia line). *λ*_exc_ = 280 nm; slit width: 2.5 nm/5 nm; Tris-HCl 50 mM, pH = 7.4.

Likewise, fluorescent adduct formation of 15 µM LE was missing when mixed with increasing aliquots of cell culture medium (without and with FCS, Fig. 6a, b, respectively). Furthermore, addition of NAC to the LE-containing medium resulted in a fluorescence burst centered at 428 nm, indicating that LE was still accessible for Michael addition. Besides diverse inorganic salts, amino acids, and vitamins, cell culture medium also contains GSH (~3 µM). This suggests that the GSH concentration is too low to observe any adduct formation with LE or that the GSH oxidation state is influenced by the interaction with other medium components.

Finally, LE was mixed directly with increasing aliquots of total cell extracts, containing biothiol concentrations higher than in the medium. An emission enhancement at 428 nm was observed (Fig. 6c), strongly indicating that some components of the cell extract form a fluorescent adduct with LE. This is in agreement with the fluorescence pattern observed in LE-exposed living cells (compare Fig. 5a). Addition of NAC to the LE-cell extract mixture did not further enhance the observed fluorescence at 428 nm, confirming that LE had already fully undergone Michael addition and could not react with the nucleophilic -SH group of NAC anymore. Of note, the band at 428 nm was specifically produced by the interaction of LE with components of the cell extract and not by the cell extract alone as confirmed by changing the order of addition in the experiment (Fig. 6d). Furthermore, as previously observed for LE with NAC in buffer solution (compare Fig. 5c), this emission band disappeared over time. As a further confirmation, we treated the cell extract with N-ethylmaleimide (NEM), which is also a strong –SH acceptor to form Michael’s adducts. As shown in Fig. 6e, addition of LE to a cell extract–NEM mixture did not result in a considerable fluorescence burst, indicating that the –SH groups were not anymore available for interaction with LE. The same fluorescence reduction was observed in buffer when NAC was preincubated with NEM before being mixed with LE (Fig. 6f, fuchsia line). Overall, these data indicate that the complex biological intracellular matrix does not inhibit fluorescent-adduct formation between LE and biothiols and its further processing to non-fluorescent derivatives.

### Impact of oxygen and GSH depletion on LE cytotoxicity and adduct stability

**Fig. 7:**
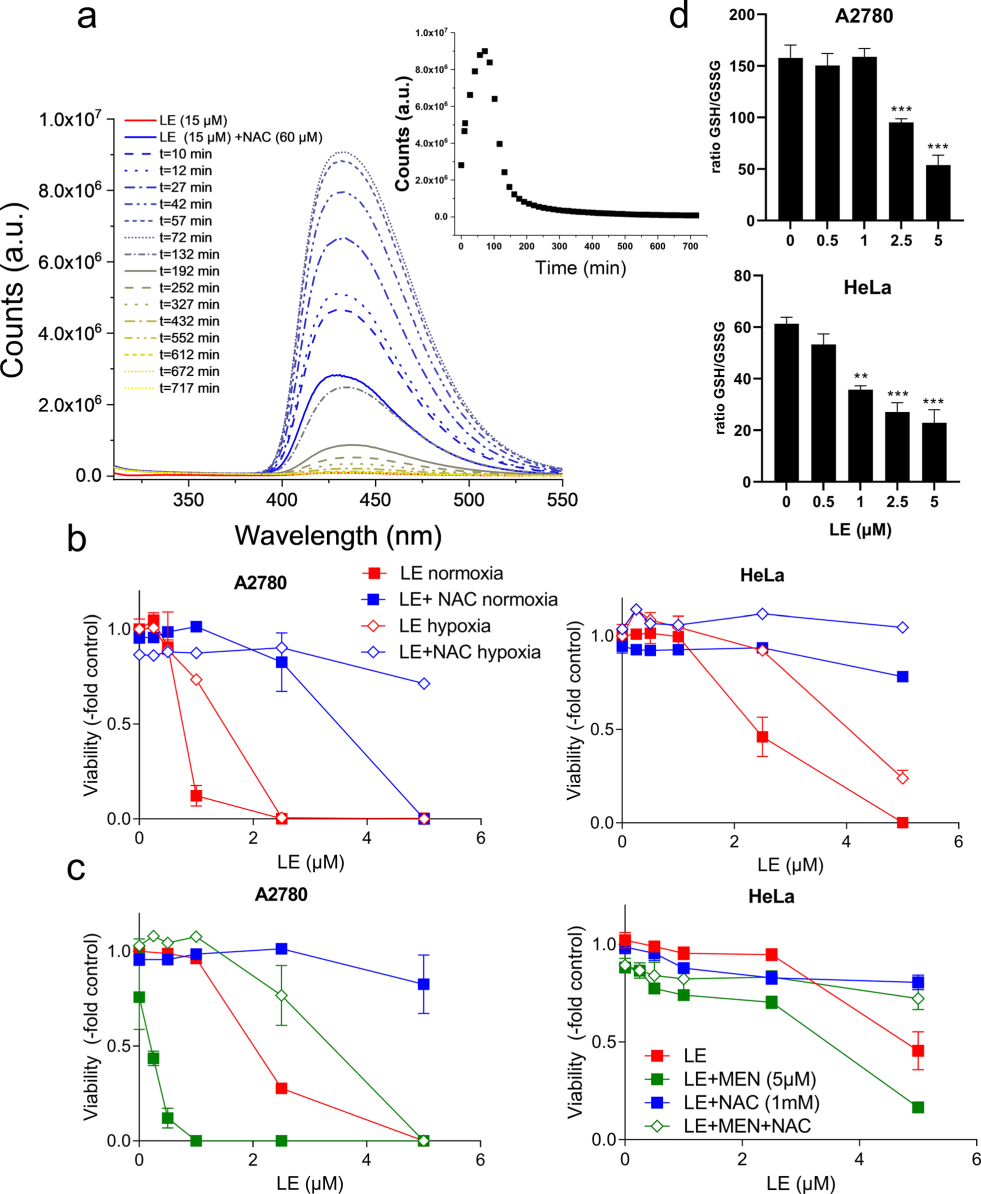
The influence of oxygen on the fluorescence and Michael’s adduct formation of LE–NAC and its cytotoxic activity. **a** Fluorescence profile over time of 15 μM LE (red solid line) in combination with 60 μM NAC in the absence of oxygen (solid and dashed lines). In the inset, fluorescence intensity of the LE + NAC emission at 428 nm vs. time. *λ*_exc_ = 280 nm; slits width: 2.5 nm/5 nm; Tris-HCl 50 mM, pH = 7.4. **b** Cell viability of indicated cell lines was measured with MTT after 72 h of LE treatment (with or without 1 mM NAC preincubation) under hypoxic and normoxic growth conditions. **c** Cell viability of A2780 and Hela cells 48 h after LE treatment with or without preincubation with MEN, NAC, or a combination of both. Each data point in (**b**) and (**c**) represents the mean ± SD of triplicate values from one representative experiment out of three delivering comparable results. All effect comparisons to the respective controls indicated significant differences by one-way ANOVA, *p* < 0.001, except in (**c**), right panel, where LE versus LE + NAC was *p* < 0.05, while LE + NAC versus LE + MEN + NAC did not significantly differ. **d** Determination of the ratio GSH/GSSG after 24 h of treatment with the indicated concentrations of LE was performed as described under “Methods”. Mean ± SD of two independent experiments performed in triplicate are shown and significance as compared with untreated controls was tested by one-way ANOVA; **p < 0.01; ***p < 0.001.

Summarizing, we discovered that LE forms a Michael’s adduct with biothiols and with components of the cell extracts. These adducts were detectable not only via mass spectrometry experiments but, more straightforwardly, through fluorescence assays (cell-free and in vitro) monitoring the emission burst at 428 nm. Nevertheless, this intense blue fluorescence disappeared over time. Considering that LE might undergo redox cycling (*vide infra*)^3^, we decided to investigate whether the fluorescence signal stability might be oxygen-dependent. Under hypoxic conditions, the fluorescence-signal was far more stable and increased overtime, reaching its maximum after 1–2 h (Fig. 7a). Then it started to slowly decrease, due to leaking of O_2_ into the cuvette over time and/or to cross-oxidation reactions that are typical of quinones^34^. Using the same experimental setting, we demonstrated that the interaction between L and NAC does not produce any fluorescence burst even under hypoxia (Supplementary Fig. 17b), indicating once again that the structural features of LE (i.e., the presence of the sugars) are necessary for the process to occur. In addition, we assessed the impact of oxygen on LE cytotoxicity in human cancer cell models. Cells, preincubated or not with NAC, were exposed to LE under normoxic or hypoxic conditions for 72 h. Interestingly, LE-treated cells preincubated with NAC were more viable under hypoxic conditions when compared with normoxic LE + NAC exposure (Fig. 7b). Thus, cell-free and in vitro results strongly suggest that the adduct between LE and the -SH group containing biomolecule is more stable under hypoxic conditions. Simultaneously, LE single treatment under hypoxic conditions also led to decreased cytotoxicity as compared with normoxia (compare Fig. 7b). Furthermore, an enhanced LE cytotoxicity was observed after combination treatment with the GSH-depleting agent MEN, indicated by a strong synergistic effect, especially in the LE-sensitive A2780 cell line (Fig. 7c). This strong synergism suggests intracellular GSH-based protection against LE cytotoxicity. Preincubation of MEN and NAC before adding LE perturbed the protective effect of NAC on LE cytotoxicity and in addition, the synergism between MEN and LE was lost (Fig. 7c). Accordingly, application of LE as a single drug significantly reduced the ratio of GSH/GSSG in both investigated cell types at concentrations exhibiting already cytotoxic activity (low µM), proving that LE, like known for MEN, intracellularly acts as GSH-depleting agent.

### Modeling of the LE–NAC adduct and its emissive properties

**Fig. 8:**
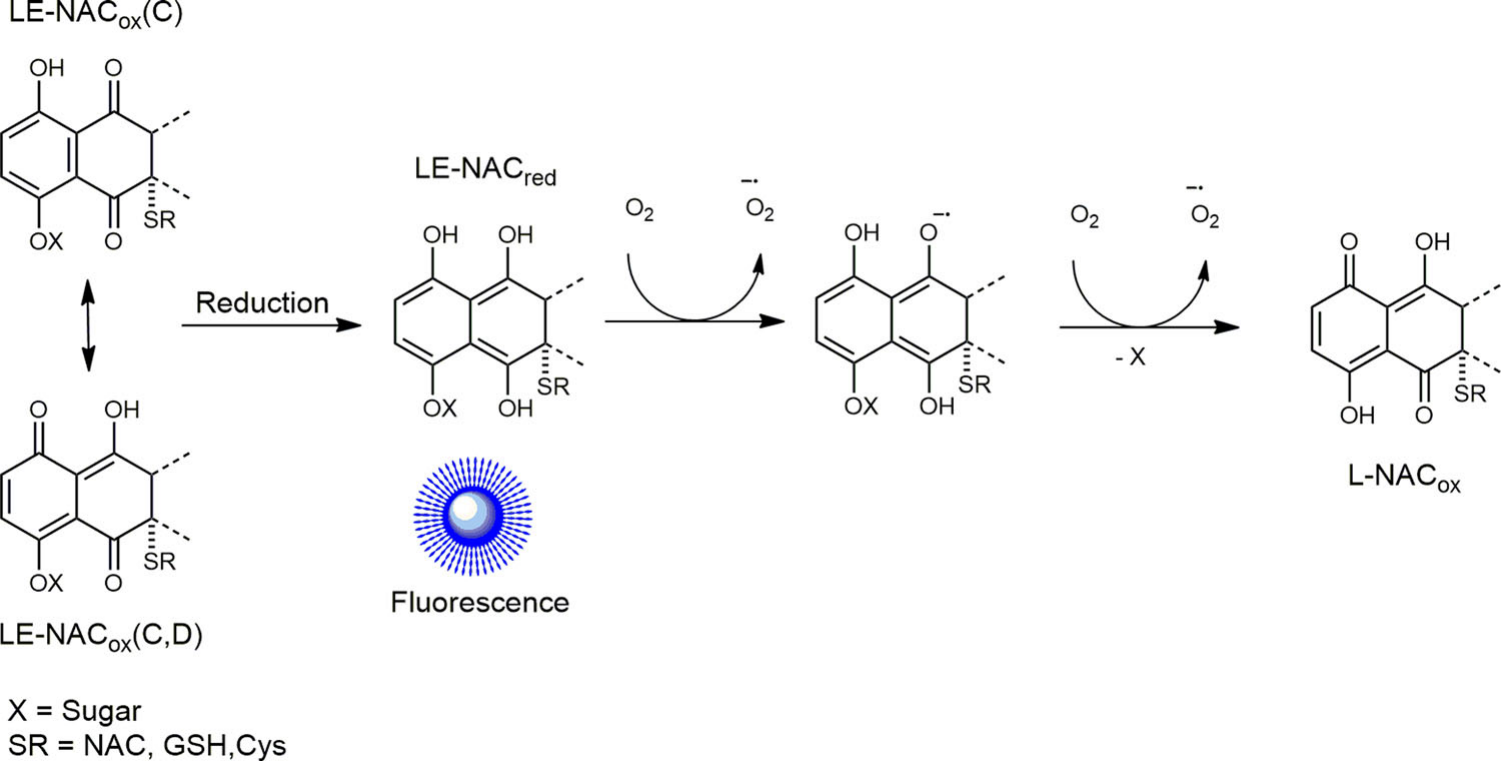
Redox process generated by the reaction of LE and NAC. Scheme illustrating how LE-NAC adduct in its oxidized state (LE–NAC_ox_) is reduced to the emissive LE–NAC_red_ species that is then oxidized by oxygen to form L-NAC_ox_.

Experimental findings indicate that the emissive adduct, generated by the reaction of LE and NAC, is involved in redox processes. This is clearly demonstrated by the oxygen dependency of the fluorescence signal observed upon addition of excess equivalents of NAC, which, also according to mass spectrometry experiments, appears to simultaneously act as a reactant and reducing agent. For this reason, we sought to gain further insights by modeling the LE–NAC adducts in their oxidized (LE–NAC_ox_) and reduced (LE–NAC_red_) forms (Supplementary Fig. 19, Supplementary Table 1 and Supplementary Data 1–3). In principle, LE–NAC_ox_ exists as two tautomeric forms: one where the quinone carbonyls are localized on the C ring, LE–NAC_ox_(C), and the other where they are localized both on the C and D rings’ LE–NAC_ox_(C,D) (Fig. 8). The LE–NAC_ox_(C,D) adduct is significantly more stable than the tautomer LE–NAC_ox_(C) according to density functional theory (DFT) optimization calculations (62.7 kcal/mol, 2.7 eV). Furthermore, the HOMO–LUMO gap of LE–NAC_ox_(C,D) is 0.68 eV (15.7 kcal/mol) smaller compared with the one determined for the LE–NAC_ox_(C) analog. These results indicate (although only qualitatively) that this species is the most easily reduced of the two, hence suggesting that the adduct undergoing the reduction process, leading to the observation of fluorescence, might be LE–NAC_ox_(C,D).

We speculate that LE-NAC_red_ (hydroquinone) is associated with the O_2_-dependent emission signal in the blue region of the spectrum (Fig. 8). Related behavior was reported for MEN, whose hydroquinone form obtained upon chemical reduction also emits in the 420-nm region^35,36^. Indeed, reduced MEN displays a π-conjugated system similar to that of the C and D rings of LE–NAC_red_. We calculated the singlet electronic transitions and the theoretical absorption spectra for this adduct using time-dependent DFT (Supplementary Fig. 20). In good agreement with the excitation profile determined experimentally (Supplementary Fig. 11), the results show that LE–NAC_red_ displays an absorption band centered at ca. 400 nm that is associated with a lowest-energy singlet state of π-π* nature, fully centered on the C and D rings of the LE-NAC adduct. As demonstrated for several quinones^34^, LE-NAC_red_ can subsequently undergo oxidation reactions through semiquinone radical intermediates, ultimately affording its fully oxidized form. In the case of LE, however, the oxidation of the hydroquinone is also associated with the loss of the sugar moiety and the generation of the nonemissive L-NAC_ox_ (as corroborated by MS, Fig. 3). The observation that the L-NAC adduct is not emissive in the experimental conditions used can be rationalized with the faster rate of oxidation of L-NAC_red_ when compared with LE–NAC_red_. As observed for other quinones^34^, this difference can also be interpreted by more efficient cross-oxidation reactions in the case of L-NAC, which lacks the hindering sugar moiety. Overall, this would explain why the onset of fluorescence is a glycosidic chain-dependent phenomenon.

## Conclusion

Within angucyclines, a family of highly bioactive polycyclic aromatic polyketides produced by *Streptomyces* species, landomycins offer unusual modes of anticancer activity^5,6^. Their characteristic landomycinone core comprises a benz[a]anthraquinone moiety with a nonaromatic B ring, while different linear glycosidic chains are characteristic for the different landomycin family members. Here we demonstrate that landomycins undergo spontaneous and highly efficient Michael adduct formation with biothiols like Cys and GSH, both under cell-free and in cellulo conditions. This chemical reaction explains the massive extracellular landomycin detoxification, e.g., by NAC. Intracellularly, potent GSH depletion—in combination with ROS induction^3^—represents a novel mode of action of landomycins and rational basis for future combination cancer-therapy approaches. The Michael addition reaction via the landomycinone core generated, strikingly in a strictly glycosidic chain-dependent fashion, an intensely fluorescent intermediate. This allowed us to follow intracellular landomycin metabolism and dissect the importance of oxygen in this process. While several synthetic fluorescence probes for biothiol detection via Michael addition reaction have been developed^37,38^, compounds of natural origin with such chemical features are comparably rare. Consequently, landomycins represent, in addition to promising anticancer GSH-depleting agents, natural-origin fluorescence probes for intracellular Michael adduct-dependent quinone metabolism.

## Methods

### Compounds

LE-overproducing *Streptomyces globisporus 1912* strain was obtained in the laboratory of B. Matselyukh (D.K. Zabolotny Institute of Microbiology and Virology, National Academy of Sciences of Ukraine, Kyiv). LE (99.5% purity, according to HPLC data) was prepared in the laboratory of J. Rohr (University of Kentucky, USA) and dissolved in absolute ethanol to obtain a 4 mg/ml stock solution. LA was isolated from *S. cyanogenus S-136* following a previously published procedure, and L was prepared by hydrolysis using formic acid^39,40^.

Menadione (MEN, Sigma Aldrich) was diluted in DMSO to obtain a 10 mM stock and stored at −20 °C. The thiol containing substances glutathione (L-GSH, ≥ 98%, Sigma Aldrich) and cysteine (L-Cys, ≥97%, Sigma Aldrich) and, in addition, the chemical GSH precursor N-acetylcysteine (NAC, ≥ 99%, Sigma Aldrich) were prior to each experiment freshly diluted in the indicated buffer or growth medium at indicated concentrations. GSH-synthesis inhibitor BSO (≥ 97%, Sigma Aldrich) was freshly diluted in ddH_2_O prior to each experiment. All the other chemicals and solvents were purchased from Sigma-Aldrich and used without further purification.

### Fluorescence spectroscopy

Fluorescence spectra were recorded using 1-cm path-length quartz cuvettes on a Horiba FluoroMax^®^−4 spectrofluorometer (Kyoto, Japan) equipped with time correlated single photon counting (TCSPC) and the data were processed using the FluorEssence v3.5 software package. Landomycin stock solutions were freshly prepared in MeOH and diluted in buffer at the indicated concentrations. Biothiol stock solutions were prepared in buffer and then diluted to the indicated concentrations. Cell extracts for spiking experiments were prepared from 1 × 10^8^ Hep3B cells in logarithmic growth phase. Cells were mechanically harvested by scraping into the growth medium, followed by centrifugation and washing three times in ice-cold phosphate-buffered saline (PBS). Then the cells were disrupted in the respective amount of buffer by five freezing/thawing cycles using liquid nitrogen. The cell lysate was centrifuged at 16,000 g for 15 min and the supernatant collected for further analysis. Total protein content was determined by the Micro BCA^TM^ kit following the instructions of the manufacturer (Thermo Scientific, Rockford, IL). Cell extract (protein content indicated in the figure caption) was spiked directly in the fluorescence cuvette where the final volume was 2 mL of 10 mM Tris-HCl, pH 7.4.

### NMR

^1^H NMR spectra were recorded at 500.10 MHz using a Bruker FT-NMR spectrometer Avance III™ 500 MHz. CD_3_OD was used as solvent to prepare landomycin and NAC solutions at the indicated concentrations.

### Mass spectrometry

Stock solutions of landomycins were prepared in methanol. These were further diluted with MilliQ water and mixed with NAC or the other biothiols (previously dissolved in water) at the desired concentrations. The resulting mixtures were diluted in ACN/MeOH 1% H_2_O and the introduction was performed via direct infusion. High-resolution spectra were recorded in the negative mode on a maXis classic (Bruker Daltonik GmbH, Bremen, Germany) hybrid ESI-Qq/oa-TOF MS instrument. Samples were diluted in ACN/MeOH 1% H_2_O and the introduction was performed via direct infusion. The following parameters were used: flow rate 3 µl/min, capillary voltage −4500 V, dry gas flow 4.0 L/min (nitrogen); dry temperature 180 °C, mass accuracy: +/−5 ppm.

### Cell culture

The human cancer cell lines Jurkat (acute T-cell leukemia, ATCC, Manassas, VA), A2780 (ovarian carcinoma, Sigma Aldrich, St. Louis, MO, US), HeLa (cervical carcinoma, ATCC), U2OS (osteosarcoma, ATCC), Hep3B (hepatoma, ATCC), and LN229 (glioblastoma, ATCC) were used during this study. Jurkat and A2780 cells were grown in RPMI-1640 (Sigma Aldrich), HeLa, Hep3B, and LN229 cells were cultured in Dulbecco’s modified eagle medium (DMEM, Sigma Aldrich). All growth media were supplemented with 10% heat-inactivated fetal bovine serum (FCS, PAA, Biowest, Nuaillé, France). Cells were passaged twice a week and regularly tested on *Mycoplasma* contamination (Mycoplasma kit, Sigma Aldrich). Cells were kept in 37 °C humid incubators containing 5% CO_2_ and 10% O_2_. For indicated experiments, the O_2_ level was reduced to 0–0.5%, creating a hypoxic environment.

### Cell viability

Cells were seeded (2 × 10^4^ cells/ml) in 100 µl of their respective growth medium per well in 96-well plates. After a recovery period of 24 h, cells were exposed to the indicated concentrations of test drugs for 72 h under normal O_2_ or hypoxic conditions. The 3-(4,5-dimethylthiazol-2-yl)-2,5-diphenyltetrazolium bromide (MTT)-based vitality assay (EZ4U, Biomedica, Vienna, Austria) was used to determine cell viability and drug synergism as published^27,28^. The effects on cell viability and drug synergism were calculated using GraphPad Prism 8.0.1 and CalcuSyn software (Biosoft, Ferguson, MO, USA), respectively. Induction of 50% cell reduction compared with untreated controls was calculated using point-to-point analysis. Data were indicated by IC_50_ values.

### Apoptosis induction measured by flow cytometry

Cells (1 × 10^5^ cells/sample) were left to recover overnight, preincubated for 24 h or 1 h with indicated thiol-containing compounds, followed by 24 h of treatment with 4 µM LE. After drug exposure, cells were collected, resuspended in annexin-V-binding buffer (10 mM HEPES, 140 mM NaCl, and 2.5 mM CaCl_2_ in 1x PBS) containing 1 µg/ml propidium iodide (PI, Sigma Aldrich) and 20 µl/ml annexin-V/APC (# 550474, Becton Dickinson (BD) Biosciences, Palo Alto, CA), and incubated for 15–20 min. Apoptosis induction was examined by flow cytometry measuring PI- and annexin-V/APC-positive and -negative cell populations (FACS Calibur, BD Biosciences). The results were analyzed using CellQuestPro software (BD Biosciences) and GraphPad Prism 8.0.1.

### LE intracellular fluorescence

The respective cells were seeded into six-well plates (CytoOne, Starblab, 1 × 10^5^/well) and allowed to settle for 24 h. Cells were treated with 4 µM LE and immediately measured by LSR Fortessa flow cytometer (BD Biosciences) in both FITC (excitation 488 nm, emission 530/30 nm) and Horizon V450 (excitation 405 nm, emission 550/50 nm) channels. LE-induced intracellular fluorescence was followed up to 6 h after treatment. Analyses were carried out using Flowing Software (University of Turku, Finland) and GraphPad Prism 8.0.1.

### Determination of GSH/GSSG ratio

The impact of drug exposure on the ratio between reduced and oxidized glutathione in cultured cells was determined by the GSH/GSSG-Glo™ assay, a luminescence-based detection system (Promega, Madison, WI). The indicated cell lines (A2780, HeLa) were seeded into 96-well plates (1 × 10^4^/100 µl/well) and, after incubation for 24 h, treated with the indicated concentration of LE added in another 100 µl of growth medium. Extraction and quantification followed exactly the instructions of the manufacturer based on the comparison with a glutathione standard curve delivered in the assay. Experiments were performed twice in triplicate.

### Live-cell imaging

Live-cell fluorescence microscopy was performed as published^28^. The indicated cell types (5 × 10^4^/well) were seeded in 8-well chambers (IBIDI, Martinsried, Germany) and left to adhere for 24 h. Afterward, the chambers were moved into the incubation chamber of a time-lapse microscope (Visitron Systems, Puchheim, Germany) and 4 µM LE added after having defined five positions for filming per well. Sequences were immediately taken with a 40x immersion oil lens in DIC and fluorescence mode using the DAPI channel (395/25 nm excitation and 460/50 nm band-pass emission filter) (VisiView software, Visitron Systems).

### Density-functional theory (DFT) optimization

The structures of the LE–NAC and L-NAC adducts were optimized at the DFT level using the lc-wpbe/6-31 + g(d,p) combination^41^. All simulations of LE were performed using one sugar monomer instead of the full moiety for reducing the computational cost. Time-dependent DFT was employed to calculate singlet–singlet transitions and the theoretical absorption spectra as previously described^42^. The nature of all stationary points was confirmed by normal-mode analysis and no imaginary frequencies were found. The solvent was modeled by means of the polarized continuum model (PCM)^43^ with water as implicit solvent. Calculations were all performed with Gaussian 16, Revision C01^44^. For analysis and visualization of computational results, the software packages GAUSSSUM 2.2 and Chimera were employed^45,46^.

### Statistics

All statistical calculations were performed in GraphPad prism software 8.0.1. The used statistical test to determine significance levels is indicated in the respective figure legends.

### Reporting summary

Further information on research design is available in the Nature Research Reporting Summary linked to this article.

## Supplementary information

Supplementary Information

Description of Additional Supplementary Files

Supplementary Data 1

Supplementary Data 2

Supplementary Data 3

Reporting Summary

## Data Availability

The authors declare that the data supporting the findings of this study are available within the article and Supplementary Information file or from the corresponding author upon reasonable request. Furthermore, the XYZ coordinates for the DFT-optimized LE–NAC_ox_(C), LE–NAC_ox_(C,D), and LE–NAC_red_ are available in Supplementary Data 1, 2 and 3, respectively.
