# Supplementary material for: Landomycins as glutathione-depleting agents and natural fluorescent probes for cellular Michael adduct-dependent quinone metabolism
Source: Commun Chem. 2021 Nov 25;4:162. doi: 10.1038/s42004-021-00600-4 (PMC9814637; doi:10.1038/s42004-021-00600-4)
Supplement: Supplementary file 1 — Supplementary Information [file 42004_2021_600_MOESM1_ESM.pdf]

**Supporting Information**

**Landomycins as glutathione-depleting agents and natural fluorescent probes for cellular Michael adduct-dependent quinone metabolism**

Alessio Terenzi, Mery la Franca, Sushilla van Schoonhoven, Rostyslav Panchuk, Álvaro Martínez, Petra Heffeter, Redding Gober, Christine Pirker, Christian R. Kowol, Rostyslav Stoika, Luca Salassa, Jürgen Rohr, Walter Berger*

**Supplementary figures**

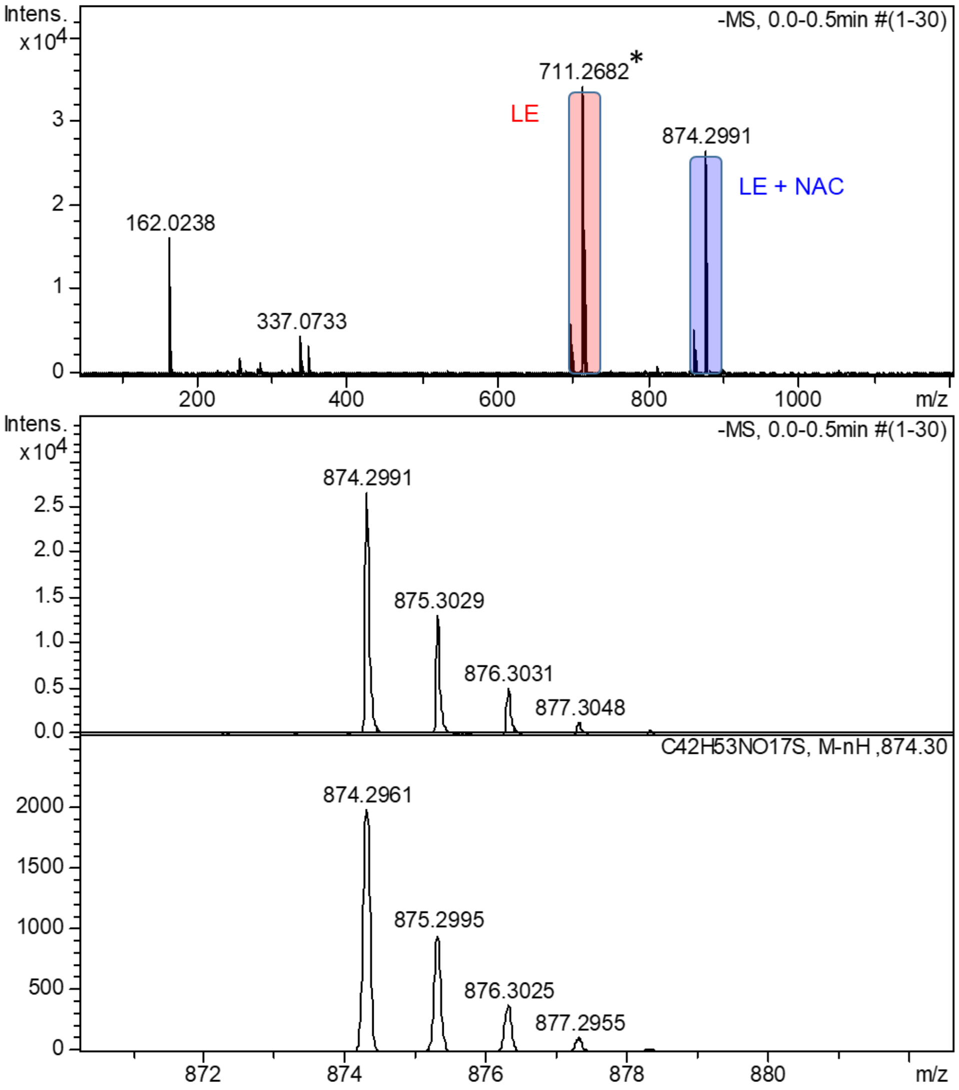


**Supplementary Fig. 1**. ESI-TOF mass spectrum of LE (0.2 mM) and NAC (0.4 mM) mixed in $H_2O$ with 20% MeOH. The m/z value at 874.3 represents LE-NAC adduct. Lower panel shows a zoom of the corresponding experimental peak (top) and a simulation of the isotopic distribution (down). The spectrum was recorded after mixing LE and NAC solutions. *The peak at 711.3, and the corresponding isotope pattern, represents the m/z of LE in its oxidized form. This peak is followed by another one at 713.3 with the isotope distribution pattern of LE in its reduced form (i.e. hydroquinone, two additional protons in mass spectrum).

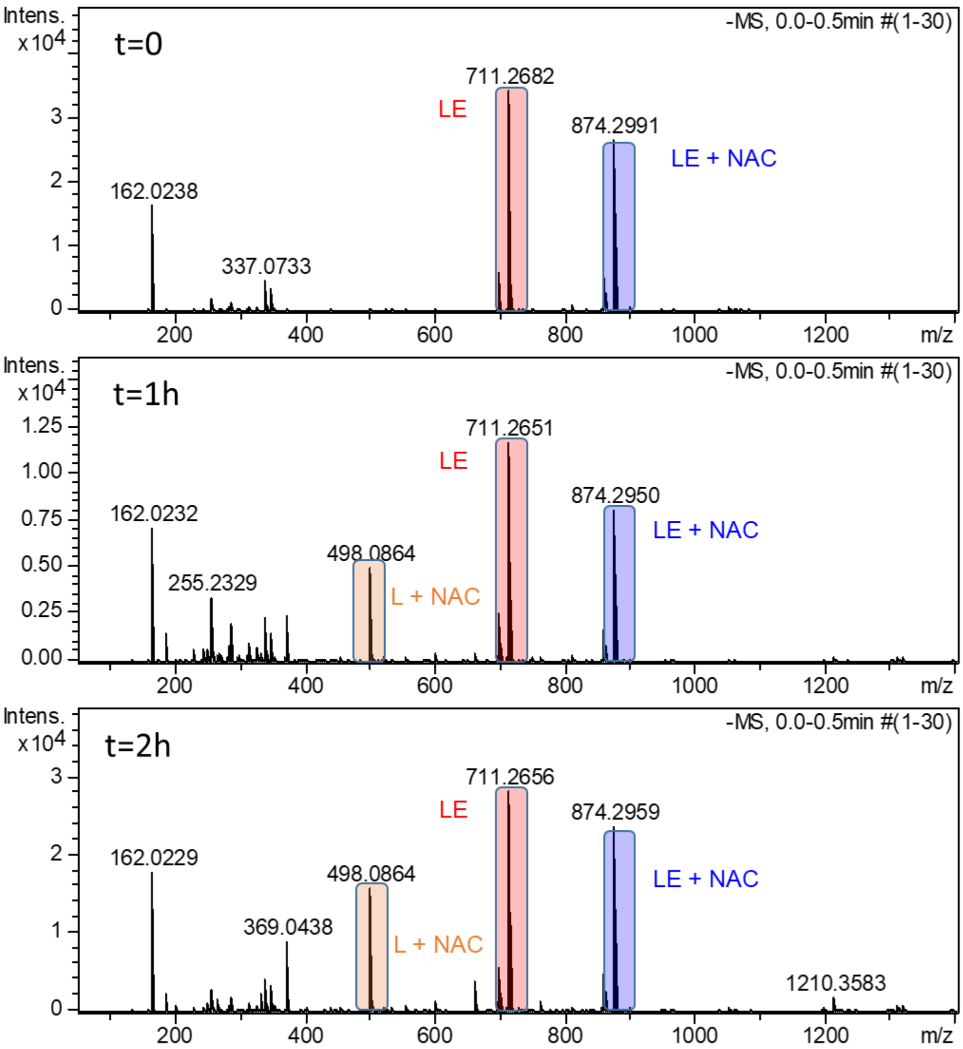


**Supplementary Fig. 2**. ESI-TOF mass spectra of LE (0.2 mM) and NAC (0.4 mM) mixed in $H_2O$ with 20% MeOH and measured at the indicated time points.

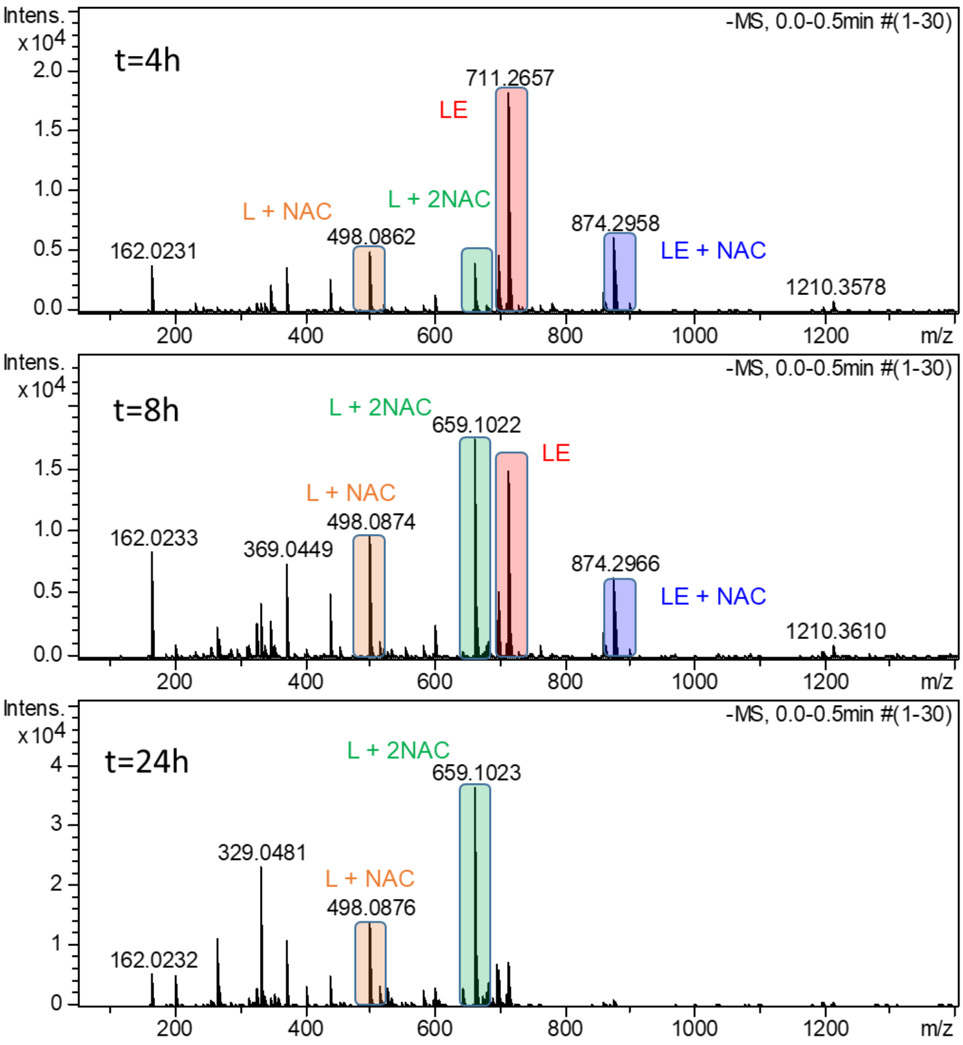


**Supplementary Fig. 3**. ESI-TOF mass spectra of LE (0.2 mM) and NAC (0.4 mM) mixed in $H_2O$ with 20% MeOH and measured at the indicated time points.

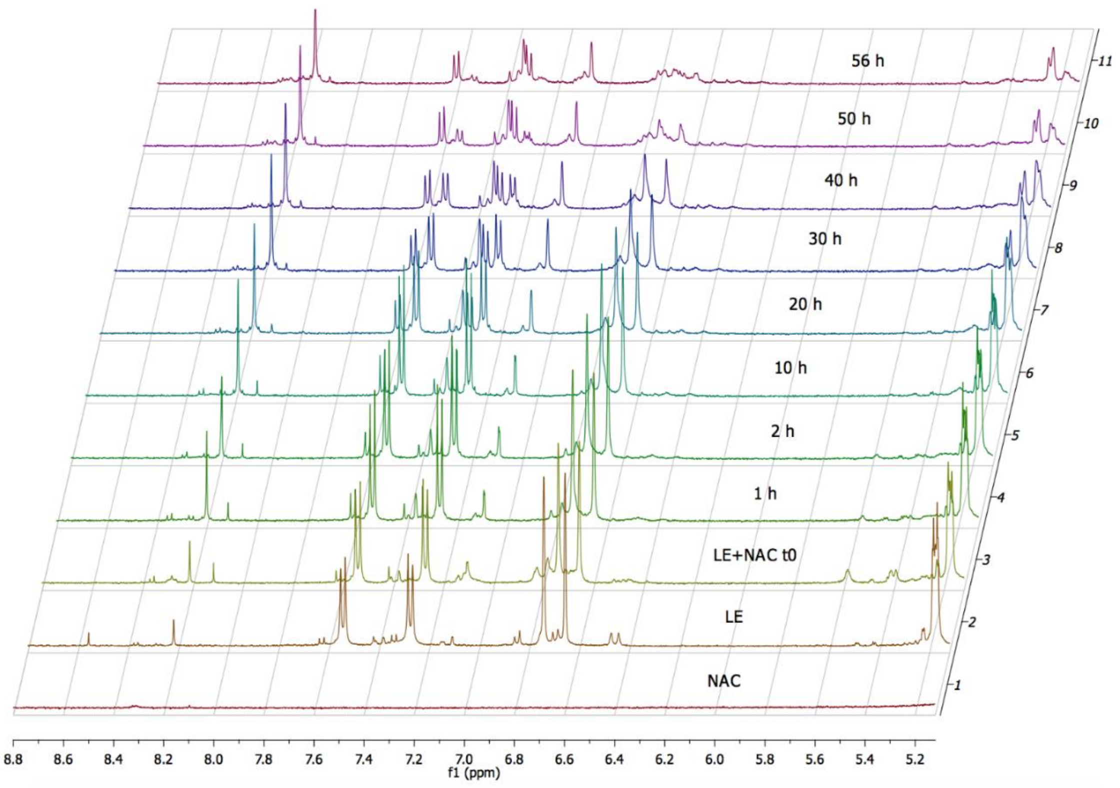


**Supplementary Fig. 4**. $^{1}H$-NMR in $CD_3OD$ LE (7 mM) mixed with NAC (10 mM) at different time points as indicated in the legend. At the bottom, $^{1}H$-NMR in $CD_3OD$ of NAC (20 mM) and LE (7 mM) as control.

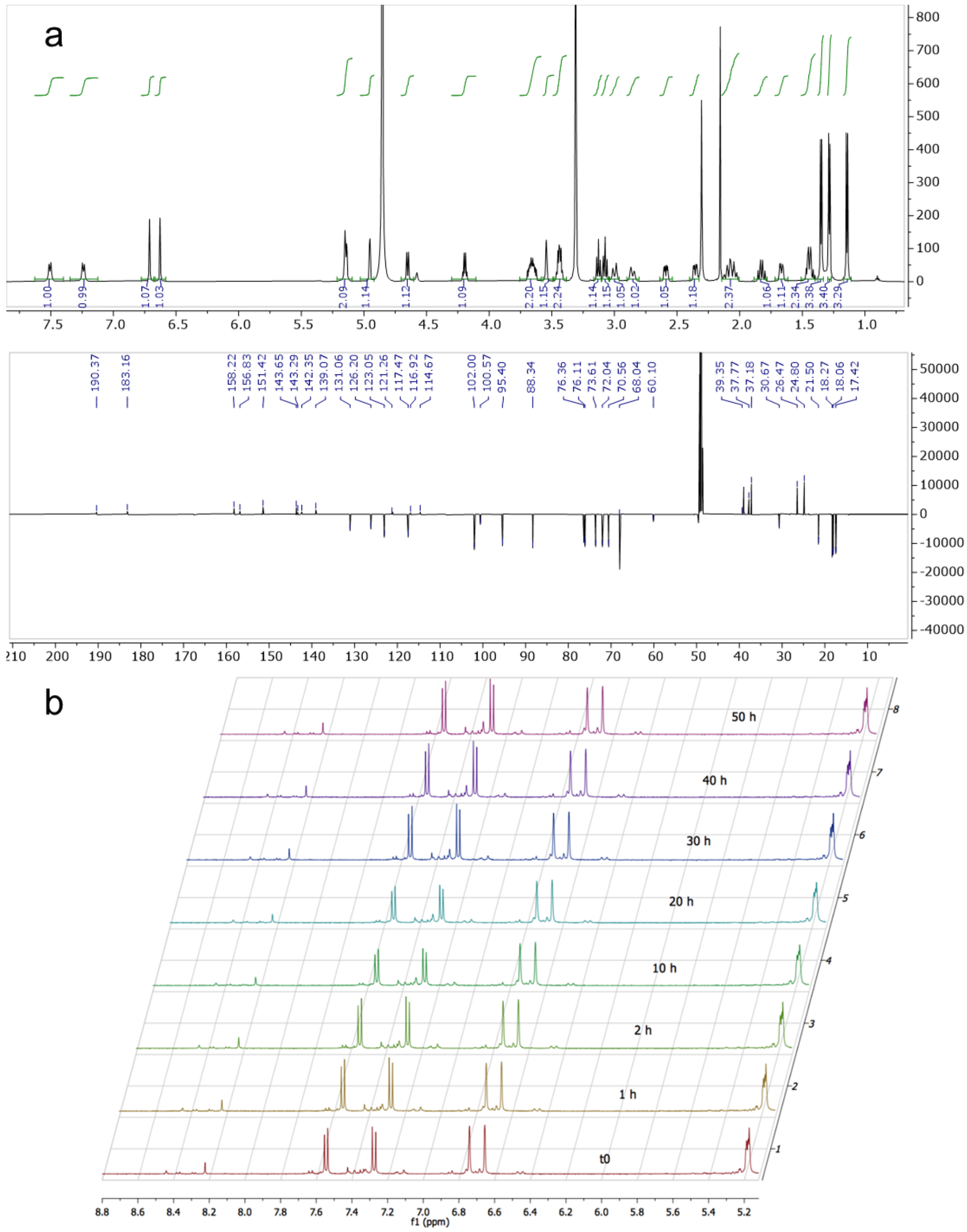


**Supplementary Fig. 5**. (a) $^{1}$H-NMR and $^{13}$C-NMR in $CD_3OD$ of LE. **$^{1}$H NMR** (600 MHz, MeOD) δ = 7.51 (d, *J* = 9.1 Hz, 1H), 7.24 (d, *J* = 9.2 Hz, 1H), 6.71 (s, 1H), 6.63 (s, 1H), 5.14 (d, *J* = 7.9 Hz, 2H), 4.95 (br s, 1H), 4.65 (d, *J* = 8.9 Hz, 1H), 4.19 (q, *J* = 6.6 Hz, 1H), 3.71–3.61 (m, 2H), 3.54 (br s, 1H), 3.44 (m, 2H), 3.13 (t, *J* = 8.8 Hz, 1H), 3.07 (t, *J* = 9.0 Hz, 1H), 3.00 (d, *J* = 16.2 Hz, 1H), 2.86 (d, *J* = 14.8 Hz, 1H), 2.59 (dd, *J* = 11.4, 5.2 Hz, 1H), 2.35 (dd, *J* = 11.8, 4.4 Hz, 1H), 2.30 (s, 3H), 2.13–2.01 (m, 2H), 1.83 (dd, *J* = 21.7, 12.0 Hz, 1H), 1.70–1.64 (m, 1H), 1.48–1.41 (m, 2H), 1.35 (d, *J* = 6.1 Hz, 3H), 1.28 (d, *J* = 6.1 Hz, 3H), 1.14 (d, *J* = 6.6 Hz, 3H). **$^{13}$C NMR** (151 MHz, $CD_3OD$) δ = 190.37 (s), 183.16 (s), 158.22 (s), 156.83 (s), 151.42 (s), 143.65 (s), 143.29 (s), 142.36 (s), 139.07 (s), 131.06 (s), 126.20 (s), 123.05 (s), 121.08 (s), 117.47 (s), 116.91 (s), 114.67 (s), 102.01 (s), 100.57 (s), 95.40 (s), 88.34 (s), 76.37 (s), 76.11 (s), 73.61 (s), 72.05 (s), 70.57 (s), 67.99 (s), 60.10 (s), 38.97 (s), 37.77 (s), 37.18 (s), 30.67 (s), 26.47 (s), 24.80 (s), 21.50 (s), 18.27 (s), 18.07 (s), 17.42 (s). (b) $^{1}$H-NMR in $CD_3OD$ LE (7 mM) at different time points as indicated in the legend.

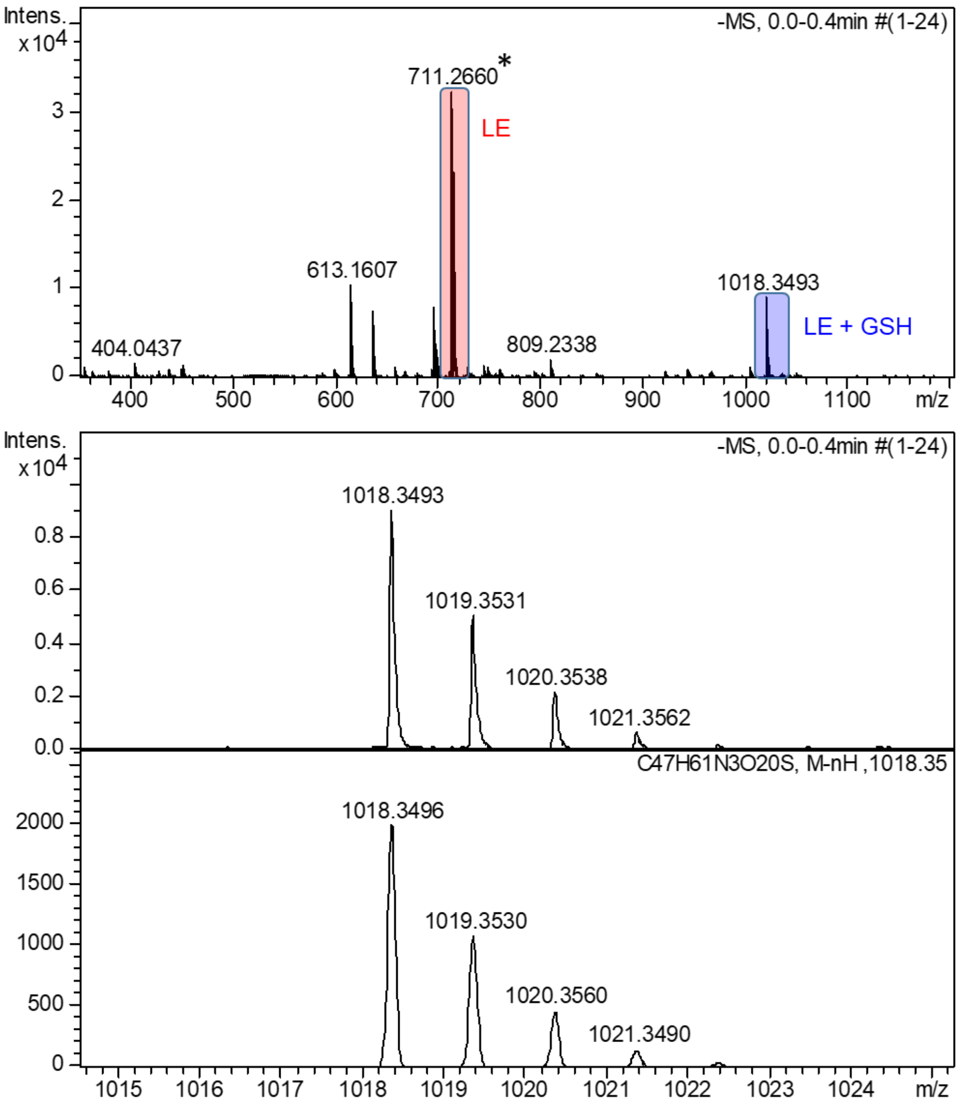


**Supplementary Fig. 6**. ESI-TOF mass spectrum of LE (0.2 mM) and GSH (0.4 mM) mixed in $H_2O$ with 20% MeOH. The m/z value at 711.3 and 1018.3 represents LE and the LE-GSH adduct, respectively. Lower panel shows a zoom of the corresponding experimental peak (top) and a simulation of the isotopic distribution (down). The spectrum was recorded after mixing LE and GSH solutions. *The peak at 711.3, and the corresponding isotope pattern, represents the m/z of LE in its oxidized form. This peak is followed by another one at 713.3 with the isotope distribution pattern of LE in its reduced form (i.e. hydroquinone, two additional protons in mass spectrum).

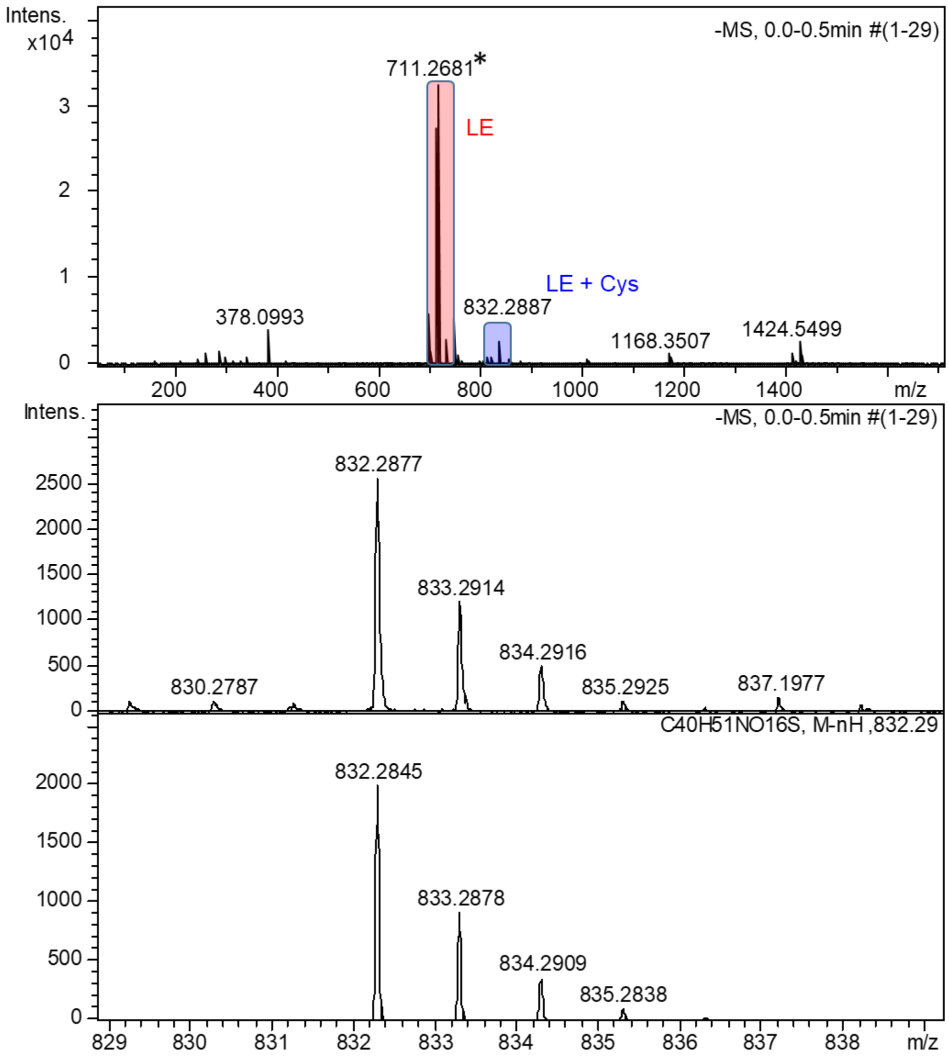


**Supplementary Fig. 7**. ESI-TOF mass spectrum of LE (0.2 mM) and Cys (0.4 mM) mixed in $H_2O$ with 20% MeOH. The m/z value at 832.3 represents LE-Cys adduct. Lower panel shows a zoom of the corresponding experimental peak (top) and a simulation of the isotopic distribution (down). The spectrum was recorded after mixing LE and Cys solutions. *The peak at 711.3, and the corresponding isotope pattern, represents the m/z of LE in its oxidized form. This peak is followed by another one at 713.3 with the isotope distribution pattern of LE in its reduced form (i.e. i.e. hydroquinone, two additional protons in mass spectrum).

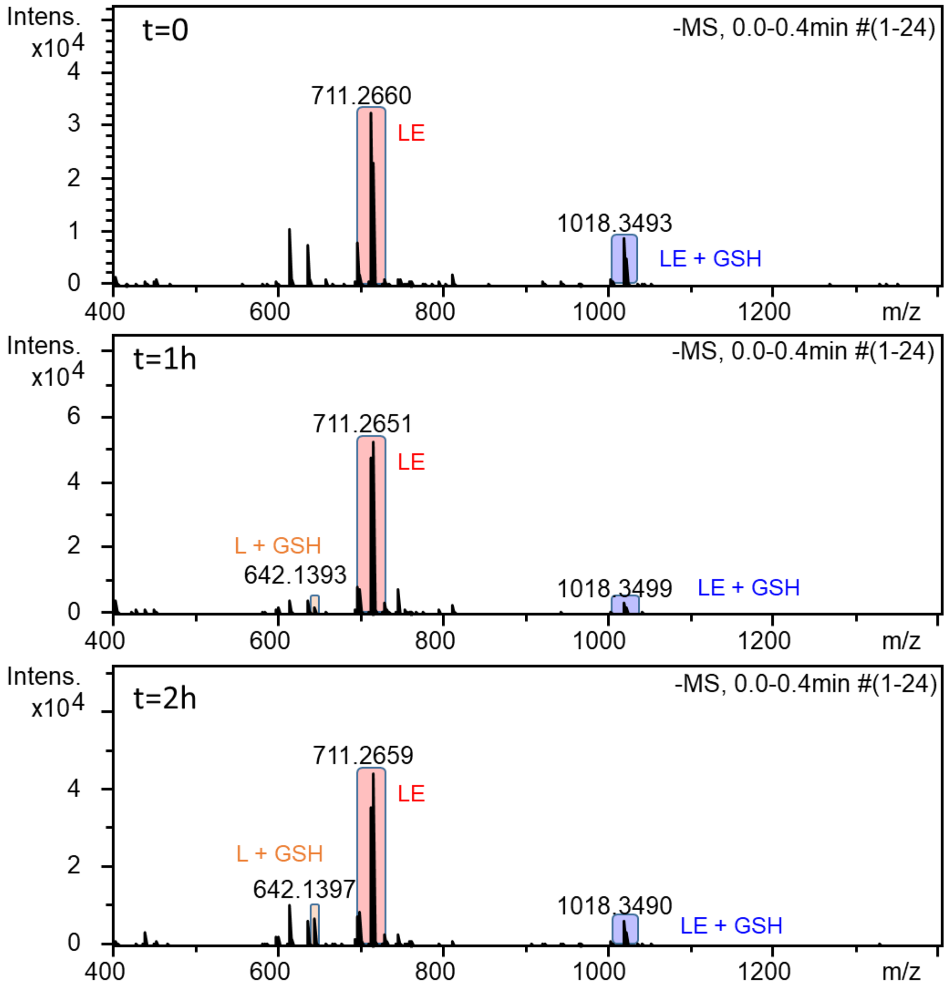


**Supplementary Fig. 8**. ESI-TOF mass spectra of LE (0.2 mM) and GSH (0.4 mM) mixed in $H_2O$ with 20% MeOH and measured at the indicated time points.

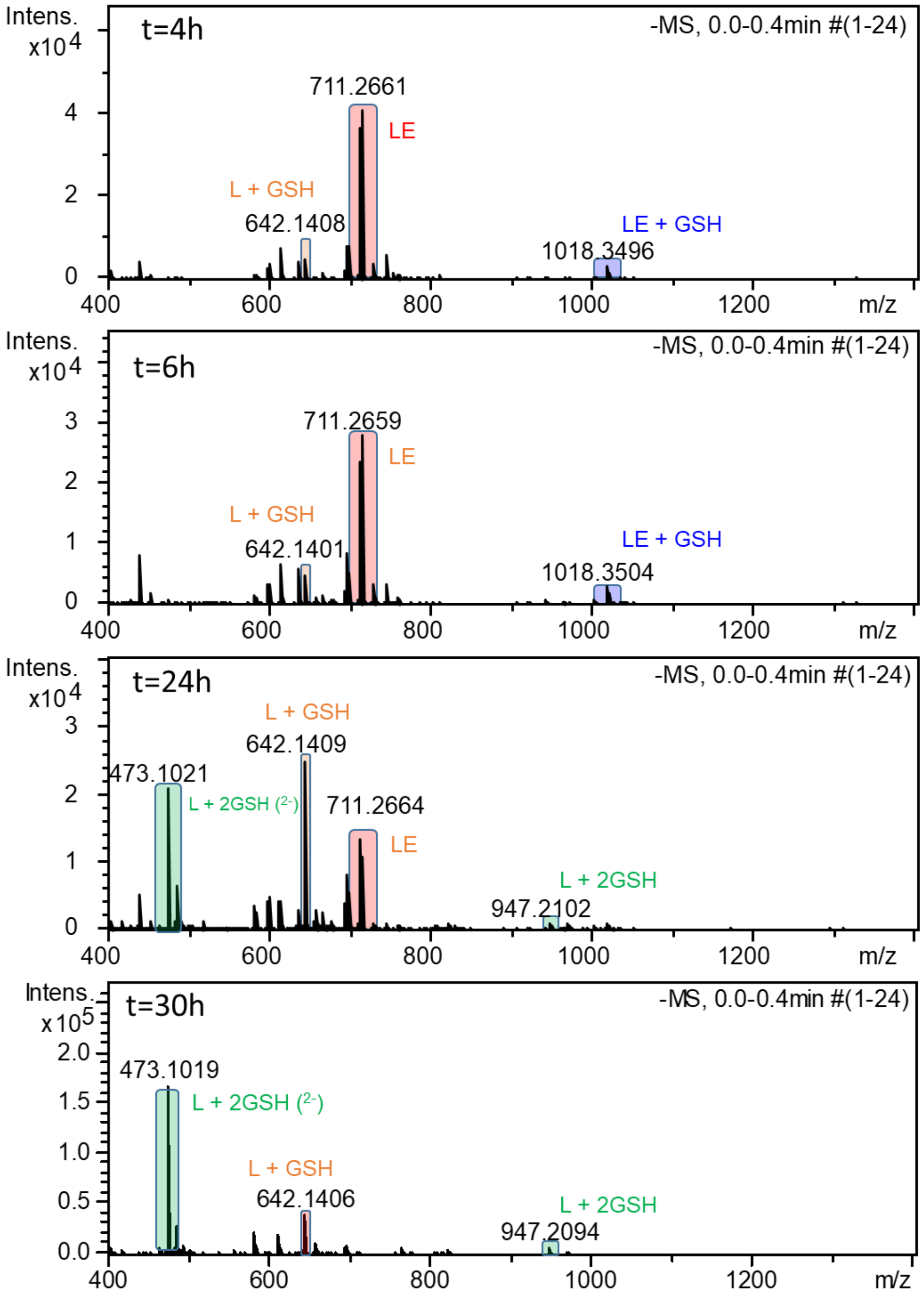


**Supplementary Fig. 9**. ESI-TOF mass spectra of LE (0.2 mM) and GSH (0.4 mM) mixed in $H_2O$ with 20% MeOH and measured at the indicated time points.

a

b

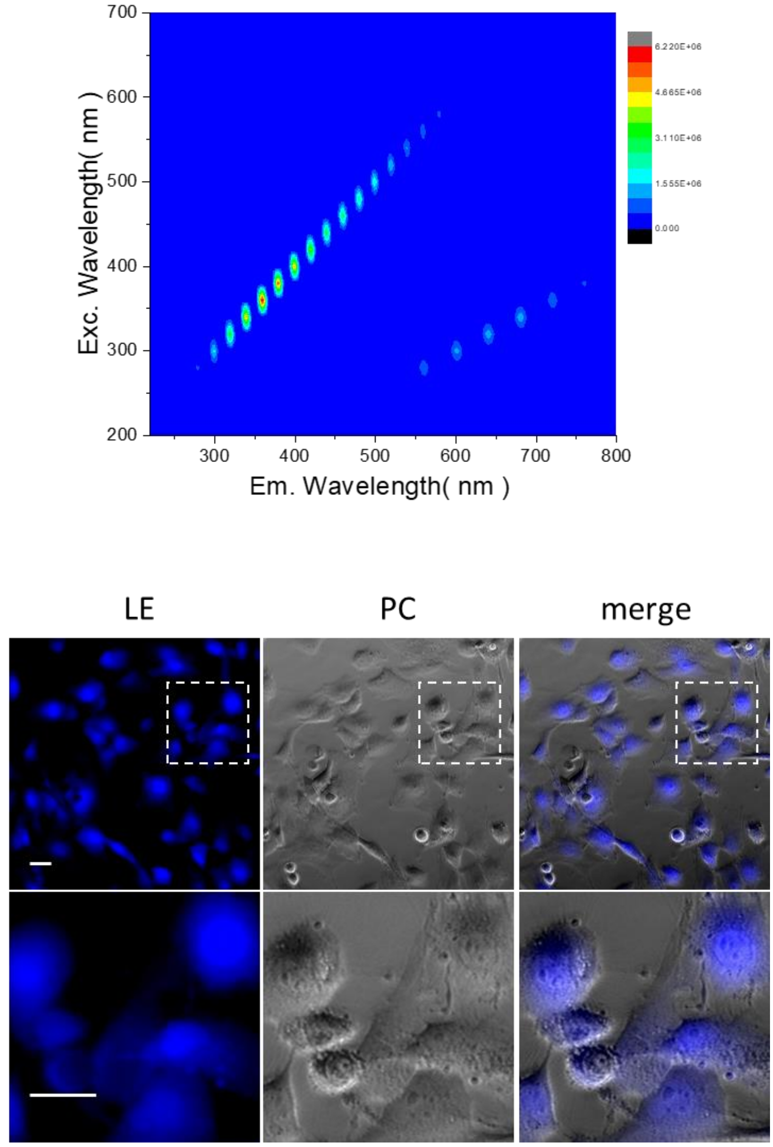


**Supplementary Fig. 10**. Cell-free and cell-based fluorescence properties of LE. (a) LE 40 µM full excitation-emission 3D landscape obtained by fluorescence spectroscopy. Fluorescence intensities are shown for excitation wavelengths from 220 nm to 700 nm (step 20 nm) while emission was measured from 235 to 800 nm. 1st and 2nd order Rayleigh scattering can be seen as diagonal ridges. Slits width: 5 nm/5nm. Buffer: Tris-HCl 50 mM, pH=7.4. (b) LE-induced intracellular fluorescence of LN229 human glioblastoma cells was followed over time by live cell imaging (DAPI channel) after treatment with 4 µM LE. The 60 min time point is shown in an overview (upper images) and the indicated region zoomed (lower images). The scale bars indicate 10 µm; PC, phase contrast.

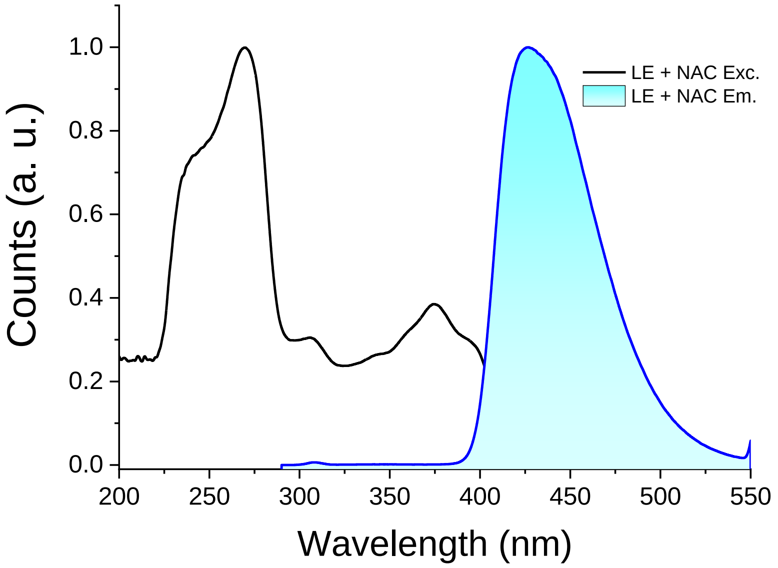


**Supplementary Fig. 11**. Fluorescence excitation/emission normalized profiles of LE in combination with 2 equivalents of NAC ($\lambda_{exc}$= 280 nm, $\lambda_{em}$= 428 nm). Slits width: 5 nm/5nm. Buffer: Tris-HCl 50 mM, pH=7.4.

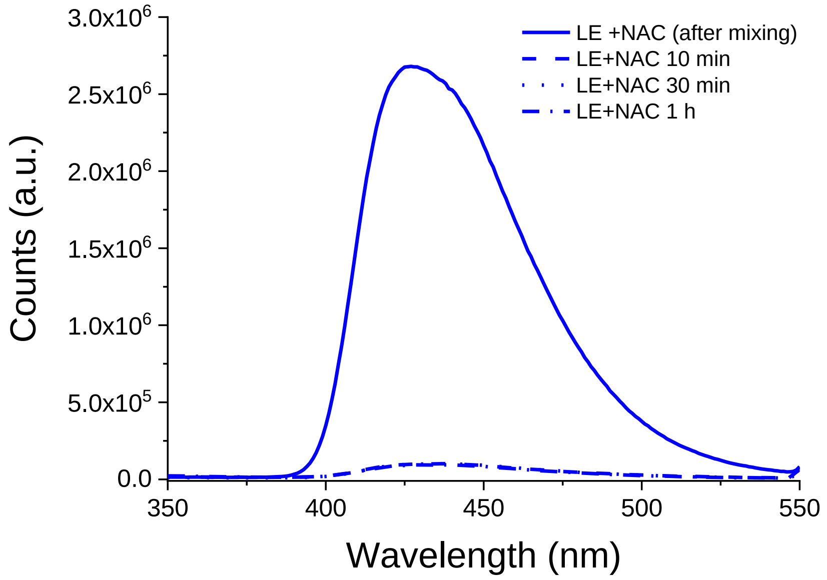


**Supplementary Fig. 12**. Fluorescence spectra of different solutions of 30 µM LE in combination with 60 µM NAC ($\lambda_{exc}$= 280 nm) recorded after the incubation time indicated in the legend. Slits width: 5 nm/5nm. Buffer: Tris-HCl 50 mM, pH=7.4

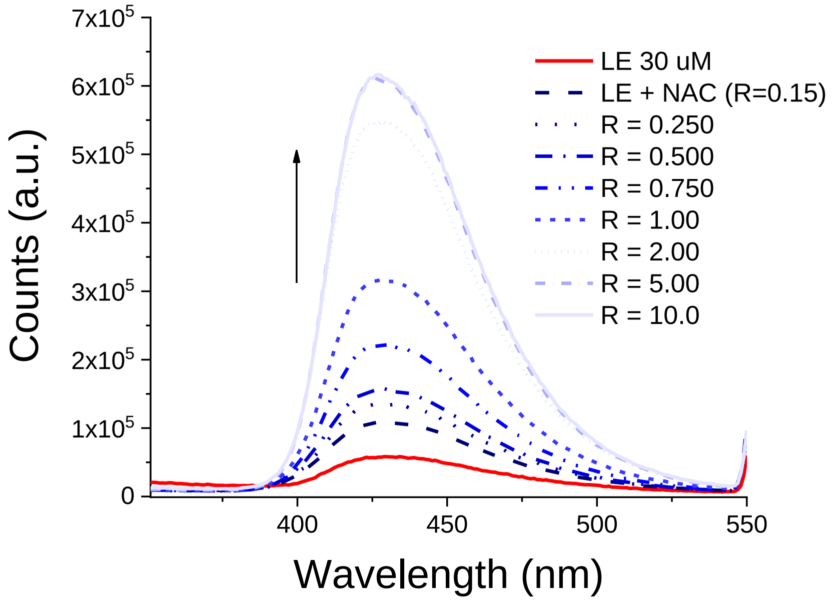


**Supplementary Fig. 13**. Fluorescence emission of LE alone and in combination with NAC ($\lambda_{exc}$= 280 nm) at different concentrations. Spectra of the solutions with different NAC/LE ratios have been recorded immediately after mixing.

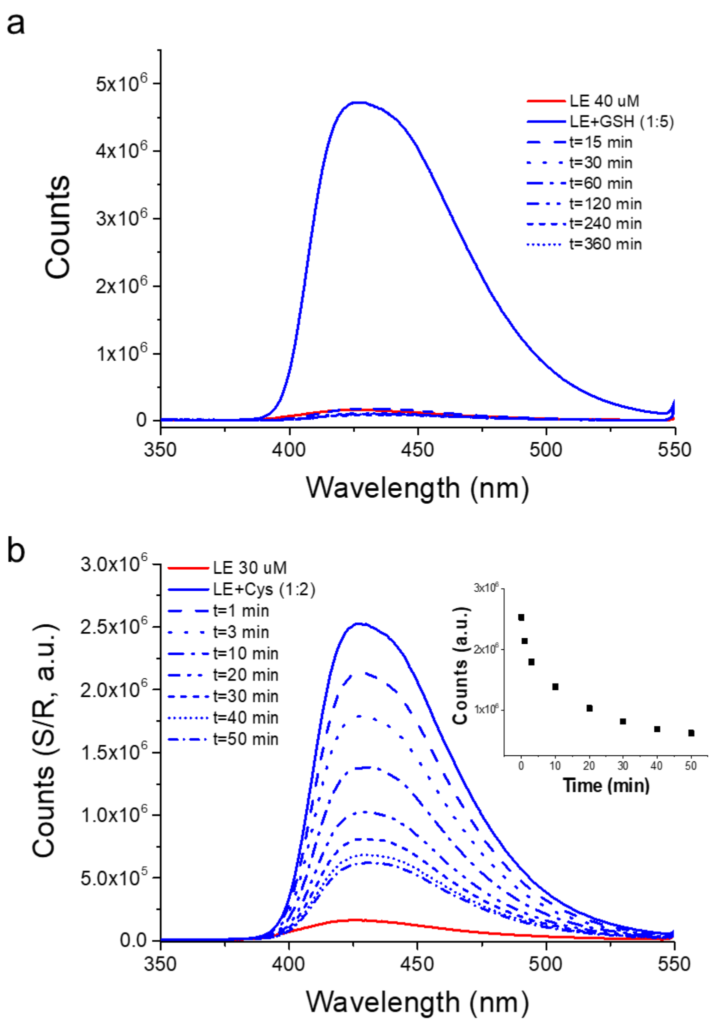


**Supplementary Fig. 14**. Fluorescence profile of LE alone and in combination with GSH (a) and with Cys (b). Spectra of the same solution have been recorded immediately after mixing LE with the biothiol (blue solid line) and after the time points indicated in the legend (blue dashed and dotted lines). $\lambda_{exc}$= 280 nm; slits width: 5 nm/5nm; Tris-HCl 50 mM, pH=7.4.

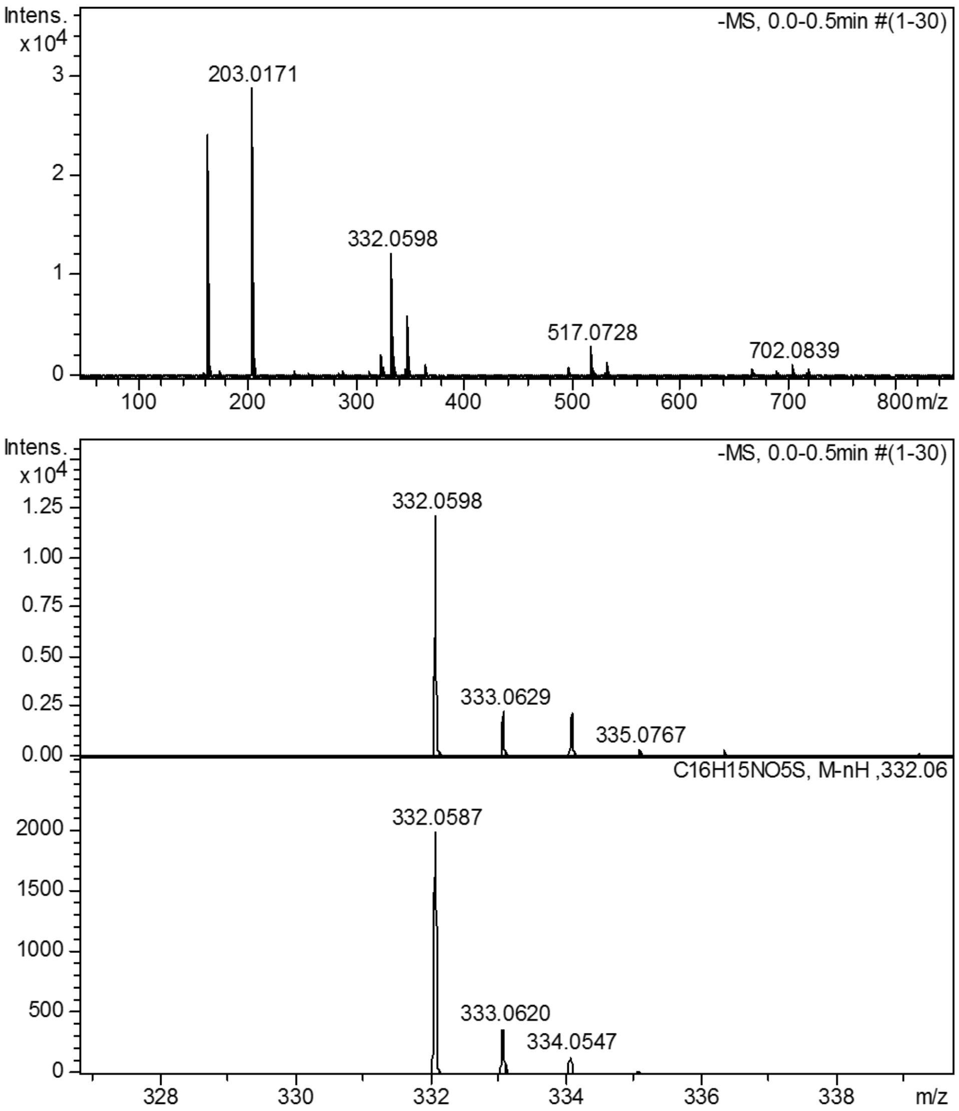


**Supplementary Fig. 15**. ESI-TOF mass spectrum of Menadione (0.2 mM) and NAC (0.4 mM) mixed in $H_2O$ with 20% MeOH. The m/z value at 203.0 and 332.1 represents Menadione (+MeOH) and the Menadione-NAC adduct, respectively. Lower panel shows a zoom of the corresponding experimental peak (top) and a simulation of the isotopic distribution (down). The spectrum was recorded after mixing Menadione and NAC solutions.

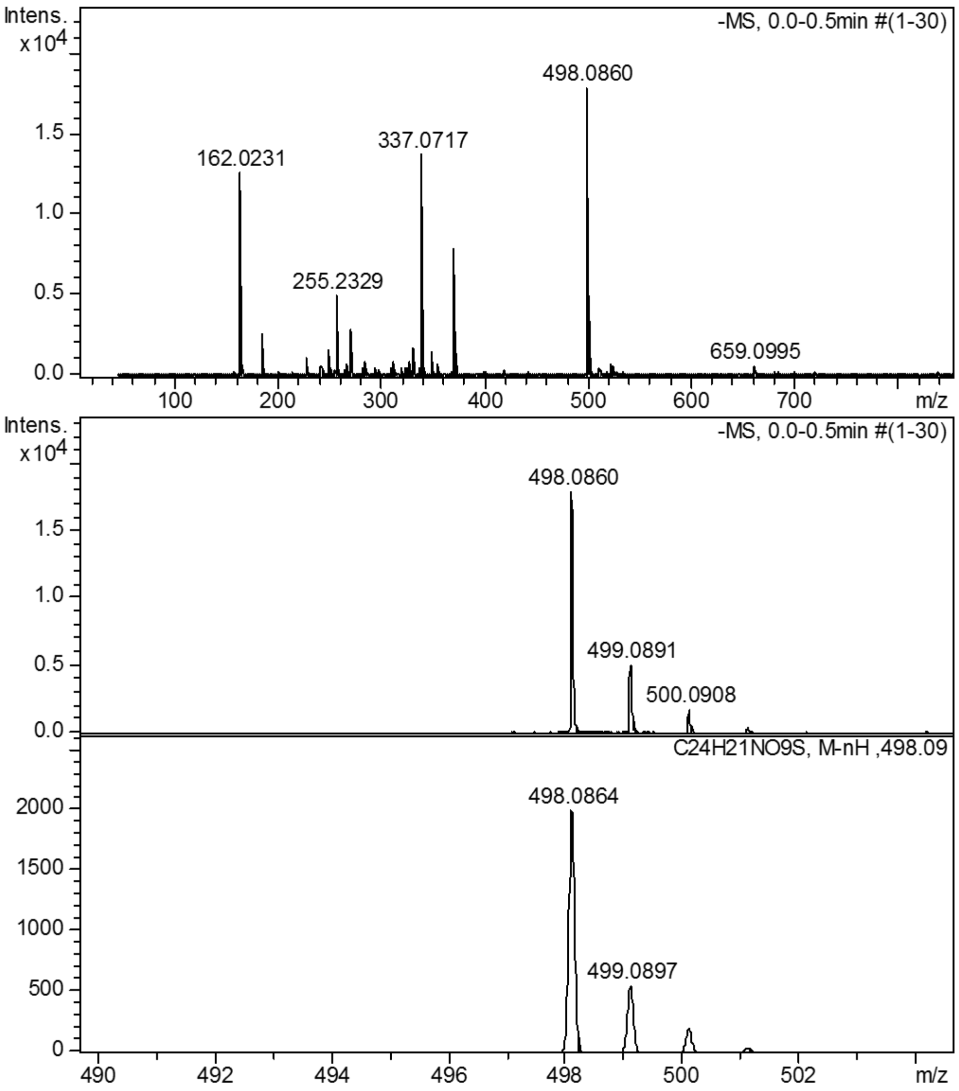


**Supplementary Fig. 16**. ESI-TOF mass spectrum of L (0.2 mM) and NAC (0.4 mM) mixed in $H_2O$ with 20% MeOH. The m/z values at 337.1 and at 498.1 represent L and L-NAC adduct in its oxidized form, respectively. Lower panel shows a zoom of the corresponding experimental peak (top) and a simulation of the isotopic distribution (down). The spectrum was recorded after mixing L and NAC solutions.

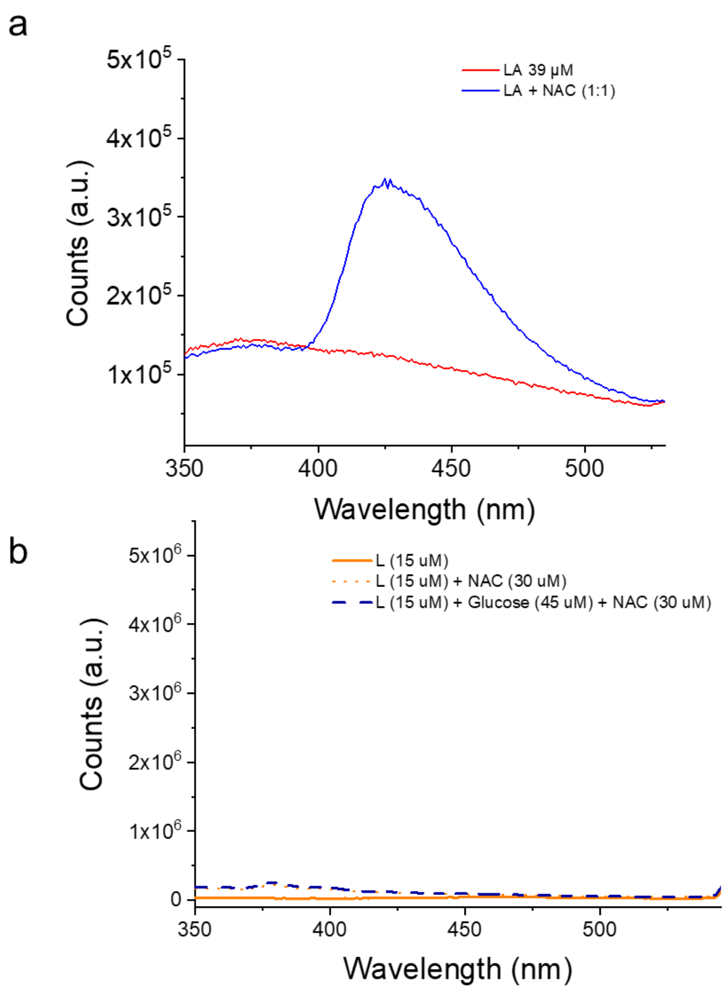


**Supplementary Fig. 17**. (a) Fluorescence profile of LA (red line) in combination with NAC (blue solid line). (b) Fluorescence profile of L (orange line) in combination with NAC (orange dotted line) or with glucose and NAC (blue dashed line), under inert atmosphere. λexc= 280 nm. Slits width: 5 nm/5nm; Tris-HCl 50 mM, pH=7.4.

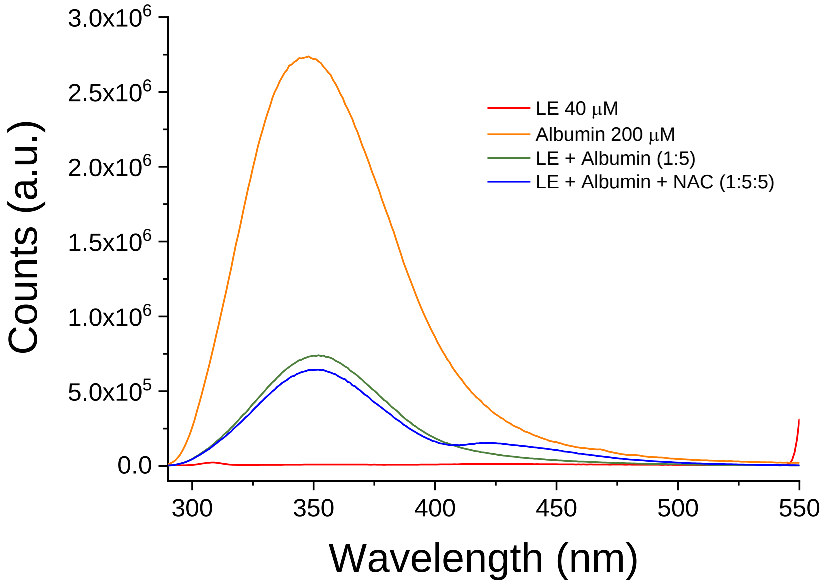


**Supplementary Fig. 18**. Fluorescence profile of LE (red), albumin (orange) and their combination (green). In blue, the fluorescence profile of a solution of LE+albumin after mixing it with NAC. $\lambda_{exc}$= 280 nm; slits width: 5 nm/5nm; Tris-HCl 50 mM, pH=7.4.

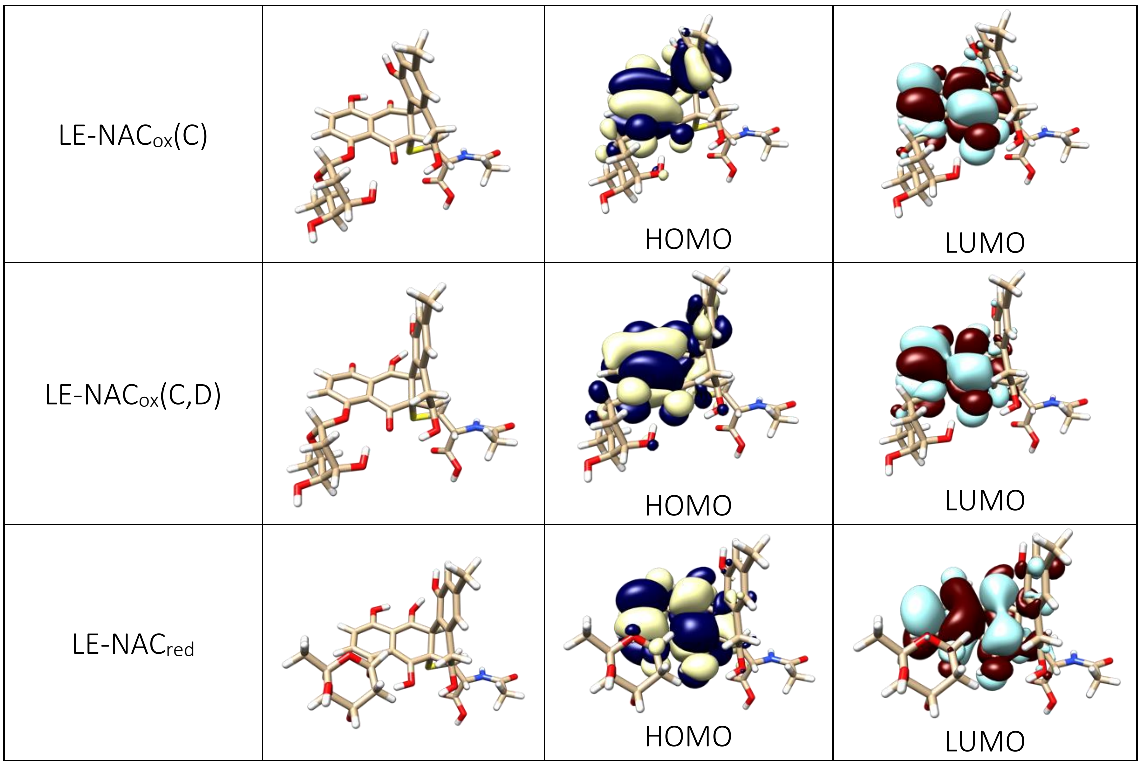


**Supplementary Fig. 19**. LE-NAC optimized structures and selected frontiers orbitals.

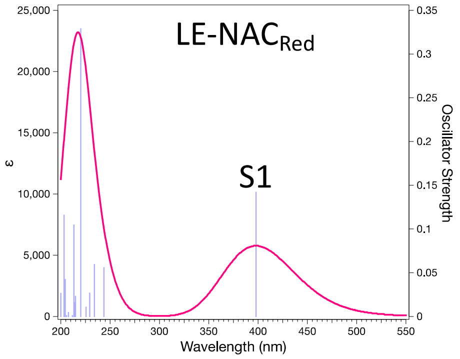


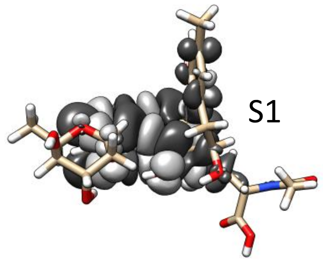


**Supplementary Fig. 20**. Calculated absorption spectrum for LE-NAC$_{red}$ in water (PCM) calculated at the lc-wpbe/6-31+g(d,p) level. Singlet–singlet transitions are shown as vertical bars with heights equal to their oscillator strengths; (Bottom) Electron density difference maps (EDDMs) for the lowest-energy singlet–singlet electronic transition (S1) of LE-NAC$_{red}$. In the EDDMs, light gray indicates a decrease in electron density, while dark gray indicates an increase.

**Supplementary Table 1**. Diagram of frontier orbital energies for the optimized LE-NAC structures.

| | **LE-NAC$_{ox}$(C)** | **LE-NAC$_{ox}$(C,D)** | **LE-NAC$_{red}$** |
|---|---|---|---|
| **Frontier orbital** | Energy (hartrees) | | |
| LUMO+5 | 0.0619667323 | 0.0614254607 | 0.0640327377 |
| LUMO+4 | 0.0607372241 | 0.0602373497 | 0.0620658290 |
| LUMO+3 | 0.0564752736 | 0.0569079181 | 0.0597121549 |
| LUMO+2 | 0.0552279534 | 0.0536726476 | 0.0587885898 |
| LUMO+1 | 0.0217929194 | 0.0172335157 | 0.0537365456 |
| **LUMO** | **-0.0266139248** | **-0.0363729268** | **0.0043673080** |
| **HOMO** | **-0.3322102340** | **-0.3169162130** | **-0.2737014380** |
| HOMO-1 | -0.3359171270 | -0.3399464680 | -0.3390243480 |
| HOMO-2 | -0.3532973250 | -0.3547368770 | -0.3513747070 |
| HOMO-3 | -0.3587936440 | -0.3586083780 | -0.3529603170 |
| HOMO-4 | -0.3762922970 | -0.3709805930 | -0.3608079090 |
| HOMO-5 | -0.3836051500 | -0.3772377590 | -0.3806730080 |
