# Supplementary material for: Landomycins as glutathione-depleting agents and natural fluorescent probes for cellular Michael adduct-dependent quinone metabolism
Source: Commun Chem. 2021 Nov 25;4:162. doi: 10.1038/s42004-021-00600-4 (PMC9814637; doi:10.1038/s42004-021-00600-4)
Supplement: Supplementary file 2 — Description of Additional Supplementary Files [file 42004_2021_600_MOESM2_ESM.pdf]

Description of Additional Supplementary Files

File Name: Supplementary Data 1

Description: XYZ coordinates for the DFT-optimized LE-NAC$_{ox}$(C)

File Name: Supplementary Data 2

Description: XYZ coordinates for the DFT-optimized LE-NAC$_{ox}$(C,D)

File Name: Supplementary Data 3

Description: XYZ coordinates for the DFT-optimized LE-NAC$_{red}$. Furthermore, we have cited them correctly in the main manuscript and in the Data Availability Statement.
