# Supplementary material for: Landomycins as glutathione-depleting agents and natural fluorescent probes for cellular Michael adduct-dependent quinone metabolism
Source: Commun Chem. 2021 Nov 25;4:162. doi: 10.1038/s42004-021-00600-4 (PMC9814637; doi:10.1038/s42004-021-00600-4)
Supplement: Supplementary file 6 — Reporting Summary [file 42004_2021_600_MOESM6_ESM.pdf]

nature research

Corresponding author(s): Walter Berger

Last updated by author(s): Oct 21, 2021

# Reporting Summary

Nature Research wishes to improve the reproducibility of the work that we publish. This form provides structure for consistency and transparency in reporting. For further information on Nature Research policies, see our Editorial Policies and the Editorial Policy Checklist.

## Statistics

For all statistical analyses, confirm that the following items are present in the figure legend, table legend, main text, or Methods section.

| n/a | Confirmed | |
|---|---|---|
| ☐ | ☒ | The exact sample size ($n$) for each experimental group/condition, given as a discrete number and unit of measurement |
| ☐ | ☒ | A statement on whether measurements were taken from distinct samples or whether the same sample was measured repeatedly |
| ☐ | ☒ | The statistical test(s) used AND whether they are one- or two-sided<br>*Only common tests should be described solely by name; describe more complex techniques in the Methods section.* |
| ☒ | ☐ | A description of all covariates tested |
| ☒ | ☐ | A description of any assumptions or corrections, such as tests of normality and adjustment for multiple comparisons |
| ☒ | ☐ | A full description of the statistical parameters including central tendency (e.g. means) or other basic estimates (e.g. regression coefficient) AND variation (e.g. standard deviation) or associated estimates of uncertainty (e.g. confidence intervals) |
| ☒ | ☐ | For null hypothesis testing, the test statistic (e.g. $F$, $t$, $r$) with confidence intervals, effect sizes, degrees of freedom and $P$ value noted<br>*Give P values as exact values whenever suitable.* |
| ☒ | ☐ | For Bayesian analysis, information on the choice of priors and Markov chain Monte Carlo settings |
| ☒ | ☐ | For hierarchical and complex designs, identification of the appropriate level for tests and full reporting of outcomes |
| ☒ | ☐ | Estimates of effect sizes (e.g. Cohen's $d$, Pearson's $r$), indicating how they were calculated |

*Our web collection on statistics for biologists contains articles on many of the points above.*

## Software and code

Policy information about availability of computer code

| | |
|---|---|
| Data collection | 1H NMR spectra were recorded at 500.10 MHz using a Bruker FT-NMR spectrometer Avance III™ 500 MHz. High resolution spectra were recorded in the negative mode on a maXis classic (Bruker Daltonik GmbH, Bremen, Germany) hybrid ESI-Qq/oa-TOF MS instrument. Flow Cytometry: Cell Quest Pro Software (BD Biosciences). Fluorescence spectra were recorded on a Horiba FluoroMax®-4 spectrofluorometer (Kyoto, Japan) |
| Data analysis | GraphPad Prism (version 8.0.1; GraphPad Software, San Diego, CA); Origin 9.5 (OriginLab Corp.); FluorEssence v3.5; MestReNova 11.0.4; ESI Compass 1.3; ImageJ 1.51f and 1.52g; Zeiss Zen 2.1 (Carl Zeiss); Zeiss Zen 2010 B SP1; For analysis and visualization of computational DFT optimization results, the software packages GAUSSSUM 2.2, and Chimera were employed. |

For manuscripts utilizing custom algorithms or software that are central to the research but not yet described in published literature, software must be made available to editors and reviewers. We strongly encourage code deposition in a community repository (e.g. GitHub). See the Nature Research guidelines for submitting code & software for further information.

## Data

Policy information about availability of data

All manuscripts must include a data availability statement. This statement should provide the following information, where applicable:

- Accession codes, unique identifiers, or web links for publicly available datasets
- A list of figures that have associated raw data
- A description of any restrictions on data availability

The authors declare that all other data supporting the findings of this study are available within the article and its Supplementary Information files, or are available from the authors upon request.

# Field-specific reporting

Please select the one below that is the best fit for your research. If you are not sure, read the appropriate sections before making your selection.

☒ Life sciences ☐ Behavioural & social sciences ☐ Ecological, evolutionary & environmental sciences

For a reference copy of the document with all sections, see nature.com/documents/nr-reporting-summary-flat.pdf

# Life sciences study design

All studies must disclose on these points even when the disclosure is negative.

| | |
|---|---|
| Sample size | All experiments were repeated independently at least three times and were performed in three technical replicates in each case to allow for statistical evaluation of data obtained. |
| Data exclusions | None of the experiments depicted data points were excluded. |
| Replication | Experiments and results were in all cases widely reproducible at least 3 times. |
| Randomization | Randomization is not applicable to this study, as all experiments depicted were performed in vitro (cell-free) or in cell culture. |
| Blinding | not applicable |

# Reporting for specific materials, systems and methods

We require information from authors about some types of materials, experimental systems and methods used in many studies. Here, indicate whether each material, system or method listed is relevant to your study. If you are not sure if a list item applies to your research, read the appropriate section before selecting a response.

## Materials & experimental systems

| n/a | Involved in the study | |
|---|---|---|
| ☒ | ☐ | Antibodies |
| ☐ | ☒ | Eukaryotic cell lines |
| ☒ | ☐ | Palaeontology and archaeology |
| ☒ | ☐ | Animals and other organisms |
| ☒ | ☐ | Human research participants |
| ☒ | ☐ | Clinical data |
| ☒ | ☐ | Dual use research of concern |

## Methods

| n/a | Involved in the study | |
|---|---|---|
| ☒ | ☐ | ChIP-seq |
| ☐ | ☒ | Flow cytometry |
| ☒ | ☐ | MRI-based neuroimaging |

## Eukaryotic cell lines

Policy information about cell lines

| | |
|---|---|
| Cell line source(s) | The human cancer cell lines Jurkat (acute T-cell leukaemia, ATCC, Manassas, VA), A2780 (ovarian carcinoma, Sigma Aldrich, St. Louis, MO, US), HeLa (cervical carcinoma, ATCC), U2OS (osteosarcoma, ATCC), Hep3B (hepatoma, ATCC) and LN229 (glioblastoma, ATCC) were used during this study. |
| Authentication | Authentication was done at the beginning of the study by STR (Eurofins Genomics) and arrayCGH (in our lab). |
| Mycoplasma contamination | Cells regularly tested on Mycoplasma contamination by PCR (Mycoplasma kit, Sigma Aldrich) |
| Commonly misidentified lines (See ICLAC register) | No misidentified lines were used. |

# Flow Cytometry

## Plots

Confirm that:

☒ The axis labels state the marker and fluorochrome used (e.g. CD4-FITC).

☒ The axis scales are clearly visible. Include numbers along axes only for bottom left plot of group (a 'group' is an analysis of identical markers).

☒ All plots are contour plots with outliers or pseudocolor plots.

☒ A numerical value for number of cells or percentage (with statistics) is provided.

## Methodology

| | |
|---|---|
| Sample preparation | Apoptosis induction measured by flow cytometry<br>Cells (1x105 cells/sample) were left to recover overnight, preincubated for 24 h or 1 h with indicated thiol- containing compounds followed by 24 h treatment with 4 µM LE. After drug exposure, cells were collected, resuspended in annexin-V-binding buffer (10 mM HEPES, 140 mM NaCl, 2.5 mM CaCl2 in 1x PBS) containing 1 µg/ml propidium iodide (PI, Sigma Aldrich) and 20 µl/ml annexin-V/APC (# 550474, Becton Dickinson (BD) Biosciences, Palo Alto, CA) and incubated for 15-20 min. Apoptosis induction was examined by flow cytometry measuring PI- and annexin-V/APC-positive and negative cell populations (FACS Calibur, BD Biosciences). Results were analysed using CellQuestPro software (BD Biosciences) and GraphPad Prism5.<br><br>LE intracellular fluorescence<br>The respective cells were seeded into six-well plates (CytoOne, Starblab; 1x105/well) and allowed to settle for 24h. Cells were treated with 4 µM LE and immediately measured by LSR Fortessa flow cytometer (BD Biosciences) in both FITC (excitation 488 nm, emission 530/30 nm) and Horizon V450 (excitation 405 nm, emission 550/50 nm) channels. LE-induced intracellular fluorescence was followed up to 6 h after treatment. Analyses were carried out using Flowing Software (University of Turku, Finland) and GraphPad Prism5. |
| Instrument | FACS Calibur, (BD Biosciences) or LSR Fortessa flow cytometer (BD Biosciences) |
| Software | CellQuestPro software (BD Biosciences) and Flowing Software (University of Turku, Finland) depending on the parameter detected. |
| Cell population abundance | Not applicable |
| Gating strategy | As drug uptake or apoptosis induction were measured, no gating strategy was necessary here. |

☐ Tick this box to confirm that a figure exemplifying the gating strategy is provided in the Supplementary Information.
